# Trace copper-mediated asexual development *via* a superoxide dismutase and induction of *AobrlA* in *Aspergillus oryzae*

**DOI:** 10.3389/fmicb.2023.1135012

**Published:** 2023-03-08

**Authors:** Takuya Katayama, Jun-ichi Maruyama

**Affiliations:** ^1^Department of Biotechnology, The University of Tokyo, Tokyo, Japan; ^2^Collaborative Research Institute for Innovative Microbiology, The University of Tokyo, Tokyo, Japan

**Keywords:** Filamentous fungi, *Aspergillus oryzae*, Copper, Conidiation, Sclerotia formation, Superoxide dismutase, *AobrlA*

## Abstract

The filamentous fungus *Aspergillus oryzae*, in which sexual reproduction remains to be discovered, proliferates mainly *via* asexual spores (conidia). Therefore, despite its industrial importance in food fermentation and recombinant protein production, breeding beneficial strains by genetic crosses is difficult. In *Aspergillus flavus*, which is genetically close to *A*. *oryzae*, structures known as sclerotia are formed asexually, but they are also related to sexual development. Sclerotia are observed in some *A*. *oryzae* strains, although no sclerotia formation has been reported in most strains. A better understanding of the regulatory mechanisms underlying sclerotia formation in *A*. *oryzae* may contribute to discover its sexual development. Some factors involved in sclerotia formation have been previously identified, but their regulatory mechanisms have not been well studied in *A*. *oryzae*. In this study, we found that copper strongly inhibited sclerotia formation and induced conidiation. Deletion of *AobrlA* encoding a core regulator of conidiation and *ecdR* involved in transcriptional induction of *AobrlA* suppressed the copper-mediated inhibition of sclerotia formation, suggesting that *AobrlA* induction in response to copper leads not only to conidiation but also to inhibition of sclerotia formation. In addition, deletion of the copper-dependent superoxide dismutase (SOD) gene and its copper chaperone gene partially suppressed such copper-mediated induction of conidiation and inhibition of sclerotia formation, indicating that copper regulates asexual development *via* the copper-dependent SOD. Taken together, our results demonstrate that copper regulates asexual development, such as sclerotia formation and conidiation, *via* the copper-dependent SOD and transcriptional induction of *AobrlA* in *A*. *oryzae*.

## 1. Introduction

*Aspergillus oryzae* is a filamentous fungus, and numerous strains of *A*. *oryzae* are industrially used for the fermentation of traditional Japanese foods, such as sake, *miso*, and soy sauce. *A*. *oryzae* is also used industrially to produce recombinant proteins (Ward, 2012) and recently expected as a host for the production of heterologous secondary metabolites (Alberti et al., 2017). Owing to its industrial importance, the establishment of crossbreeding techniques *via* sexual development is expected to be useful in generating beneficial strains. However, sexual reproduction remains to be discovered in *A*. *oryzae* (Wada et al., 2012), and it proliferates through asexual cycle, in which genetically identical individuals are generated. Therefore, the discovery of sexual reproduction in *A*. *oryzae*, which generates genetically diverse strains, would greatly enhance its potential industrial applications.

Asexual filamentous fungi, such as *A*. *oryzae*, mainly proliferate *via* asexual spores called conida. The regulation of conidiation has been well established in *Aspergillus nidulans*. Conidiation is initiated by the transcriptional induction of the gene for the transcription factor *brlA*, sequentially activating the downstream genes for transcription factors *abaA* and then *wetA*, which are required for proper conidiation (Park and Yu, 2012). These components of the conidiation regulatory pathway are conserved and are also required for conidiation in *A*. *oryzae* (Ogawa et al., 2010). In addition to conidiation, several filamentous fungal species belonging to Ascomycota and Basidiomycota asexually develop a hardened mycelial aggregation called the sclerotium, which survives for long periods under unfavorable environmental conditions (Georgiou et al., 2006; Dyer and O’Gorman, 2012). In some *Aspergillus* species, the sclerotium is closely related to the formation of sexual reproductive structures (Kwon-Chung and Sugui, 2009), and functions as a repository for sexual reproductive structures in *Aspergillus flavus*, which is genetically close to *A*. *oryzae* (Horn et al., 2009; Gibbons et al., 2012). Therefore, the sclerotium would be expected to play a crucial role in sexual reproduction of *A*. *oryzae*, if it exists. However, no sclerotia formation has been reported in most *A*. *oryzae* industrial strains (Murakami, 1971), and understanding of its regulatory mechanisms will be required to enhance sclerotia formation, which probably contributes to the discovery of sexual reproduction of *A*. *oryzae*.

Unlike conidiation, the knowledges about the regulatory mechanism of sclerotia formation are limited. However, several environmental factors have been shown to be involved in sclerotia formation, such as temperature, light, oxygen availability, humidity, pH, and medium composition (Dyer and O’Gorman, 2012). Oxidative stress caused by reactive oxygen species (ROS) plays a crucial role in sclerotia formation (Georgiou et al., 2006). Sclerotia formation in *A*. *oryzae* is also known to be affected by environmental conditions; malt extract medium has been used to induce sclerotia formation and potato dextrose medium has been used to induce conidiation (Nakamura et al., 2016). In addition, AoRim15 which is homologous to Rim15, a stress-responsive kinase in *Saccharomyces cerevisiae*, is involved in sclerotia formation in *A*. *oryzae* (Nakamura et al., 2016), suggesting that certain environmental stresses are involved in sclerotia formation. Although the involvement of oxidative stress-responsive pathways in sclerotia formation has been well studied (Georgiou et al., 2006), little is known about the genetic basis underlying the regulation of sclerotia formation. In *A*. *oryzae*, two transcription factors, EcdR and SclR, are involved in sclerotia formation (Jin et al., 2011a,b). Deletion of *ecdR* stimulates sclerotia formation and inhibits conidiation (Jin et al., 2011a). In contrast, deletion of *sclR* leads to loss of the ability to from sclerotia and dense conidial formation (Jin et al., 2009, 2011b). However, the pathways regulating sclerotia formation that these transcription factors act upon remain unclear. In addition, these phenotypes caused by deletion of *ecdR* and *sclR* suggest the relationship between the regulatory mechanisms of sclerotia formation and conidiation.

Copper functions as a cofactor for some enzymes and participates in quite diverse cellular processes, including respiration (cytochrome *c* oxidase), detoxification of ROS (superoxide dismutase (SOD); (Ruiz et al., 2021), nitrogen utilization (nitrite reductase; Long et al., 2015), iron uptake (ferroxidase; Schrettl et al., 2004), and biosynthesis of secondary metabolites (laccase; Upadhyay et al., 2013). In some *Aspergillus* species, copper is known to be required for conidial pigmentation (Chang et al., 2019). Moreover, genetic evidence for the involvement of copper in asexual development such as conidiation and sclerotia formation has been established in filamentous fungi. Deletion of *Afmac1*, encoding a copper-binding transcription factor, results in a conidiation defect in *Aspergillus fumigatus* (Kusuya et al., 2017). The copper transporter BcCcc2 is required for sclerotia formation in *Botrytis cinerea* (Saitoh et al., 2010). In addition to this genetic evidence, the effects of copper treatment on sclerotia development have also been reported. Copper has been suggested to inhibit sclerotia formation in the *A*. *oryzae* G15 strain (Long et al., 2017) and in an *Aspergillus* strain (Rogers and Li, 1985). In contrast, copper induces sclerotia formation in *Penicillium thomii* (Zhang et al., 2014; Zhao et al., 2014). These findings support the conclusion that copper is involved in the asexual development of filamentous fungi. However, high concentrations of copper (> 80 μM) used in these studies possibly cause toxic effects such as oxidative stress (Zhang et al., 2014; Zhao et al., 2014), and physiological effects of copper on asexual development have not been investigated.

In this study, we found that trace amounts of copper (1.6 μM) strongly induced conidiation and inhibited sclerotia formation in *A*. *oryzae*, and demonstrated that sclerotia formation is inhibited by AoBrlA, which is critical for conidiation. In addition, the copper-dependent SOD AoSod1 functions in induction of copper-mediated conidiation and in inhibiting of sclerotia formation. These results suggest that activation of SOD by copper leads to the stimulation of *AobrlA*, resulting in the induction of conidiation and inhibition of sclerotia formation.

## 2. Materials and methods

### 2.1. Strains and growth conditions

The *A*. *oryzae*, *Aspergillus sojae*, and *Aspergillus luchuensis* strains used in this study are listed in Supplementary Table S1. Yeast extract-glucose (YG) medium (5 g/L yeast extract and 10 g/L glucose) and malt extract (ME) medium [20 g/L malt extract (ORIENTAL YEAST Co., Ltd., Tokyo, Japan), 20 g/L glucose, and 1 g/L HIPOLYPEPTON (FUJIFILM Wako Pure Chemical Co., Osaka, Japan)] were used for the growth of *A*. *oryzae*. To investigate the effects of metal ions, 0.1% trace elements solution (30.56 mM ZnSO_4_·7H_2_O, 1.60 mM CuSO_4_·5H_2_O, 0.36 mM FeSO_4_·7H_2_O, 0.67 mM MnSO_4_·4H_2_O, 0.26 mM Na_2_B_4_O_7_·10H_2_O, and 0.04 mM (NH_4_)_6_Mo_7_O_24_·4H_2_O; Rowlands and Turner, 1973) and each component of the trace elements solution were added. To investigate the effect of copper, 0.1% CuCl_2_ solution (1.60 mM) was added. To test growth, conidiation, and sclerotia formation, conidial suspensions (1 × 10^4^/5 μL) were spotted onto the agar medium and incubated in the dark at 30°C. The Δ*ecdR*, Δ*AobrlA*, Δ*AoabaA*, and Δ*AowetA* mutants hardly formed conidia, and the mycelial mass instead of the conidial suspension was inoculated onto the agar medium.

### 2.2. Transformation of *Aspergillus oryzae*

Classical transformation and transformation using genome editing of *A*. *oryzae* were performed as previously described (Maruyama and Kitamoto, 2011; Katayama et al., 2019). Dextrin-peptone-yeast extract (DPY) medium (20 g/L dextrin, 10 g/L polypeptone, 5 g/L yeast extract, 5 g/L KH_2_PO_4_, and 0.5 g/L MgSO_4_·7H_2_O) or dextrin-peptone (DP) medium (20 g/L dextrin, 10 g/L polypeptone, 5 g/L KH_2_PO_4_, and 0.5 g/L MgSO_4_·7H_2_O) were used for pre-culture. Czapek-dox (CD) medium (3 g/L NaNO_3_, 2 g/L KCl, 1 g/L KH_2_PO_4_, 0.5 g/L MgSO_4_·7H_2_O, 0.002 g/L FeSO_4_·7H_2_O, and 20 g/L glucose [pH 5.5]) was used to select the transformants. For selection using the pyrithiamine-resistant *ptrA* marker, 0.1 μg/ml pyrithiamine was added. To remove the genome-editing plasmid from the transformants, dextrin was added to CD medium instead of glucose. To positively select *sC* and *niaD* mutants, the selenate medium (3 g/L NaNO_3_, 2 g/L KCl, 1 g/L KH_2_PO_4_, 0.5 g/L MgCl_2_, 20 g/L glucose, 30 mg/L d-methionine, and 9.5 mg/L sodium selenate [pH 5.5]) and the chlorate medium (1.31 g/L leucine, 2 g/L KCl, 1 g/L KH_2_PO_4_, 0.5 g/L MgSO_4_·7H_2_O, 0.002 g/L FeSO_4_·7H_2_O, 20 g/L glucose, 0.015 g/L methionine, and 57.6 g/L KClO_3_ [pH 5.5]) were used, respectively. For the growth of *niaD* mutants, 3 g/L NH_4_Cl was added to CD medium instead of NaNO_3_. For the growth of *sC* mutants, 0.015 g/L methionine was added to CD medium. For the growth of *pyroA* mutants, 0.5 mg/L pyridoxine hydrochloride was added.

### 2.3. DNA manipulation

*Escherichia coli* DH5α strain was used for DNA manipulation. Polymerase chain reaction (PCR) for plasmid construction was performed using KOD-Plus-Neo (TOYOBO, Osaka, Japan). The In-Fusion HD Cloning kit (TaKaRa Bio, Ohtsu, Japan) and the seamless ligation cloning extract (SLiCE) method (Okegawa and Motohashi, 2015a,b) were used for plasmid construction. Genomic PCR was performed using KOD FX Neo (TOYOBO). Primers used in this study are listed in Supplementary Table S2.

### 2.4. Total DNA extraction from *Aspergillus oryzae* strains and southern blot analysis

Total DNA extraction from *A*. *oryzae* strains and Southern blot analysis were performed as previously described (Maruyama and Kitamoto, 2011).

### 2.5. Construction of plasmids generating control strains for genetic modification

The *U6* promoter with a target sequence for *ku70* deletion was amplified from pRGE-gwAup (Katayama et al., 2019) using SmaIIF1-PU6-F2nd/gku70-PU6R primers and ligated with *Sma*I-digested pRGE-gRT6 (Katayama et al., 2019), yielding a genome-editing plasmid pRGE-gku70. The upstream and downstream flanking regions of *ku70* were amplified from RIB40 genomic DNA using 19IF-ku70-5F/ku70-5R and ku70-3F/19IF-ku70-3R primer sets, respectively. These fragments were ligated with *Bam*HI-digested pUC19 (TaKaRa Bio), yielding the donor plasmid pΔku70. The RIB40Δku5–2 strain was constructed by co-introducing pRGE-gku70 and pΔku70 into RIB40. Deletion of *ku70* and no unexpected integration of the donor plasmid were confirmed by Southern blot analysis (Supplementary Figure S1A). The genome-editing plasmid was then removed from the Δ*ku70* strain by subculturing on the CD medium containing dextrin instead of glucose.

The R40KS strain was constructed from RIB40Δku5–2 by introducing *Not*I-digested psCΔ5 (Katayama et al., 2021). The *sC*^−^ mutants were selected on the selenate medium as *sC*^−^ mutants exhibit selenate resistance (Yamada et al., 1997), and the deletion of the upstream and 5′ regions of *sC* was confirmed by genomic PCR (Supplementary Figure S1B).

The R40KSN strain was constructed from the R40KS strain by introducing a DNA fragment amplified from CDK1 (Katayama et al., 2021) genomic DNA using DniaD-F/DniaD-R primers. The *niaD*^−^ mutants were selected on chlorate medium as *niaD*^−^ mutants exhibited chlorate resistance (Ishi et al., 2005), and the deletion of the 3′ and downstream regions of *niaD* was confirmed by genomic PCR (Supplementary Figure S1C).

The *U6* promoter with a target sequence for *pyroA* deletion was amplified from pRGE-gwAup using SmaIIF1-PU6-F2nd/gpyroA-PU6R primers and ligated with *Sma*I-digested pRGE-gRT6, yielding the genome-editing plasmid pRGE-gpyroA. The upstream and downstream flanking regions of *pyroA* and the *pyrG* marker were amplified from RIB40 genomic DNA using 19IF-pyroA5F/pyrG-pyroA5R, pyrG-pyroA3F/19IF-pyroA3R, and pyrGF/pyrGR primer sets, respectively. These DNA fragments were ligated into *Bam*HI-digested pUC19, yielding pΔpyroA-pyrG. To remove the *pyrG* marker from pΔpyroA-pyrG, the plasmid DNA was digested with *Xho*I and self-ligated to yield pΔpyroA. Strain R40KSNP was constructed by co-introducing pRGE-gpyroA and pΔpyroA into the R40KSN strain. The *pyroA* deletion was confirmed using genomic PCR (Supplementary Figure S1D). The genome-editing plasmid was then removed from the transformant by subculturing on the CD medium containing dextrin instead of glucose.

### 2.6. Construction of gene deletion strains

For gene deletion, the approximate 1-kb flanking regions of the target gene were amplified from RIB40 genomic DNA using the primers listed in Supplementary Table S2. These fragments were then integrated into *Bam*HI-digested pUC19 together with the *pyroA* marker amplified from RIB40 genomic DNA using AopyroAF/R primers. The DNA fragment for gene deletion was amplified from the resulting plasmid and introduced into the R40KSNP strain. Deletion of the target gene was confirmed by genomic PCR (Supplementary Figure S2).

### 2.7. Construction of complemented strains

To reintroduce *AobrlA* into the *AobrlA* deletion mutant, *AobrlA* gene was integrated into its native locus, where its ORF was replaced with the *pyroA* marker. For plasmid construction, the upstream region and ORF of *AobrlA* were amplified from RIB40 genomic DNA using brlA-1/sC-brlA-R primers. The *sC* marker was amplified from RIB40 genomic DNA using sC-F/pyroA-sC-R primers. The 5′ region of the *pyroA* marker was amplified from RIB40 genomic DNA using AopyroAF/19IF-pyroAmid-R primers. These fragments were ligated with *Bam*HI-digested pUC19. The DNA fragment amplified from the yielding plasmid using brlA-1/19IF-pyroAmid-R primers was introduced into the *AobrlA* deletion mutant. Integration of the introduced DNA fragment into the *AobrlA* locus was confirmed using genomic PCR (Supplementary Figure S3A).

For complementation of *sC*, *Not*I-digested pisC (Mamun et al., 2020) was introduced into the *sC*^−^ strains, and the integration of the introduced DNA fragment into the *sC* locus was confirmed by genomic PCR.

To reintroduce *Aosod1* and *AoccsA* into the corresponding deletion mutants, their promoter, ORF, and terminator regions were amplified from RIB40 genomic DNA using the primers listed in Supplementary Table S2, and then ligated with *Xho*I-digested pUXN (Mori et al., 2019). The resulting plasmids were digested with *Not*I and introduced into the corresponding deletion mutants. Integration of the introduced DNA fragment into the *niaD* locus was confirmed using genomic PCR (Supplementary Figures S3B,C).

For complementation of *niaD*, *Not*I-digested pUXN was introduced into the *niaD*^−^ strains, and integration of the introduced DNA fragment into the *niaD* locus was confirmed by genomic PCR.

### 2.8. Transcriptional analysis

Mycelia were collected from agar plates and homogenized using a multibead shocker (Yasui Kikai, Osaka, Japan). Total RNA was extracted from the homogenized mycelia, and cDNA synthesis and qRT-PCR were performed as described previously (Katayama et al., 2019). Primers used for qRT-PCR are listed in Supplementary Table S2.

### 2.9. Stereomicroscopy

Stereomicroscopy was performed using a stereomicroscope (VB-G25/VB-7010; Keyence Co., Ltd., Osaka, Japan).

### 2.10. Statistical analysis

The results of three independent experiments are shown as mean values, and error bars represent standard deviation (SD), as indicated in the figure legends. Statistical significance was tested using a two-sample Student’s *t* test in Microsoft Excel, and the results are indicated as *p < 0.05, or **p < 0.01. Tukey-Cramer multiple comparison was performed using software “R” version 4. 0. 3, with a significance level (*p* < 0.05).

## 3. Results

### 3.1. Trace copper induces conidiation and suppresses sclerotia formation

**Figure 1:**
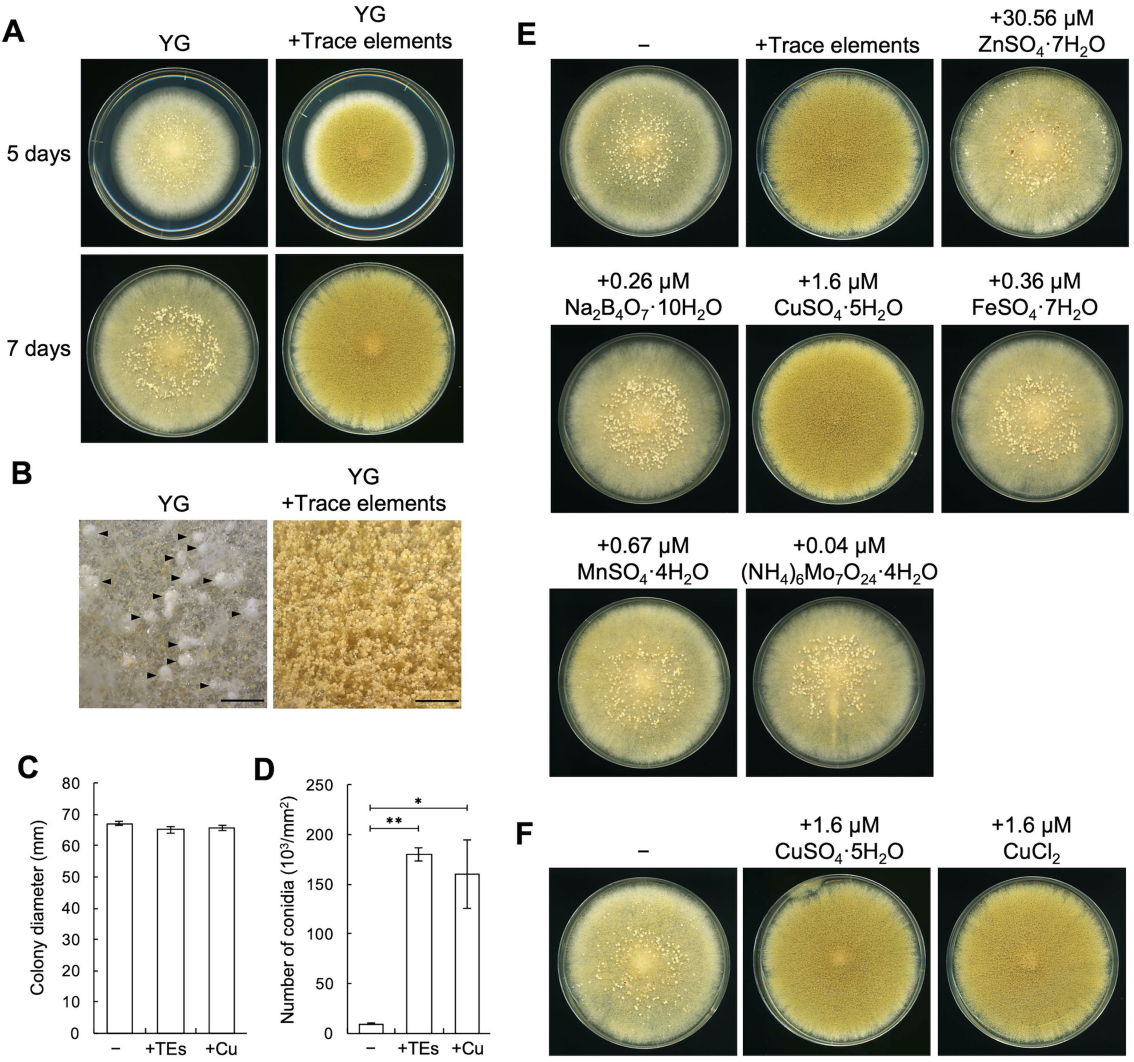
Copper induces conidiation and suppresses sclerotia formation. **(A)** Conidial suspensions (1 × 10^4^/5 μL) of the RIB40 strain were inoculated onto YG agar media with or without trace elements solution and incubated in dark at 30°C for 5 or 7 days. Sclerotia were observed as the white mass structures around the center of the colonies. **(B)** Stereomicroscopic images of the colonies shown as 5 days in panel **A**. Arrowheads indicate sclerotia. Bars: 2 mm. **(C,D)** Effect of the trace elements solution and CuSO_4_·5H_2_O on the growth and conidiation efficiency. Conidial suspensions (1 × 10^4^/5 μL) of the RIB40 strain were inoculated onto YG (−), YG containing trace elements solution (+TEs), and YG containing 1.6 μM CuSO_4_·5H_2_O (+Cu), and then incubated in dark at 30°C for 5 days. Colony diameter **(C)** and number of conidia **(D)** were measured. Data are shown as means ± SD of three independent experiments. *p < 0.05, **p < 0.01 by Student’s *t*-test. **(E)** Conidial suspensions (1 × 10^4^/5 μL) of the RIB40 strain were inoculated onto YG agar media containing the indicated metals and incubated in dark at 30°C for 7 days. **(F)** Conidial suspensions (1 × 10^4^/5 μL) of the RIB40 strain were inoculated onto YG agar media containing the indicated metals and incubated in dark at 30°C for 7 days.

To explore the conditions under which sclerotia formation is induced, we incubated the sclerotiogenic *A*. *oryzae* RIB40 strain (Murakami, 1971) on various agar media and found that numerous sclerotia were formed on the YG agar medium (Figure 1A). Stereomicroscopic observation also showed sclerotia formation and sparsely formed conidiophores on the YG agar medium (Figure 1B). In contrast, sclerotia formation was hardly detected, and dense conidiophore formation was observed on YG agar medium supplemented with the trace elements solution, which are often used to prepare media for *Aspergillus nidulans* (Fukuda et al., 2009; Figures 1A,B). Supplementation with trace elements solution significantly increased the conidiation efficiency without affecting growth (*p* < 0.01; Figures 1C,D). These results suggest the involvement of a component(s) of the trace elements solution in asexual development of *A*. *oryzae*. When the components of the trace elements solution were separately added to the YG medium, supplementation with 1.6 μM CuSO_4_·5H_2_O was found to strongly inhibit sclerotia formation and induce conidiation without affecting the growth, similar to what was observed upon supplementation with the trace elements solution (Figures 1C–E). Supplementation with 1.6 μM CuCl_2_ had indistinguishable effects on development (Figure 1F). These results indicate that trace copper affects asexual development on YG agar medium. To investigate the effects of copper on media other than YG medium, CuSO_4_·5H_2_O was added to ME agar medium, on which sclerotia formation is induced (Nakamura et al., 2016). Sclerotia formation was also inhibited on this medium, and conidiation was induced by supplementing CuSO_4_·5H_2_O to the ME agar medium as well as YG agar medium (Supplementary Figure S4). Taken together, these results indicate that trace copper induces conidiation and inhibits sclerotia formation in *A*. *oryzae*.

### 3.2. Copper affects asexual development in *Aspergillus oryzae* industrial strains, *Aspergillus sojae*, and *Aspergillus luchuensis*

To investigate the generality of the copper-mediated effects on asexual development, *A*. *oryzae* industrial strains and other *Aspergillus* species, such as *Aspergillus sojae* and *Aspergillus luchuensis* were incubated on YG agar media with or without copper. In the sclerotiogenic *A*. *oryzae* TK-32 and TK-38 strains, numerous sclerotia were formed on the YG agar medium, whereas copper supplementation inhibited sclerotia formation and strongly induced conidiation, as observed in the RIB40 strain (Supplementary Figures S5A,B). The *A*. *oryzae* TK-41, *A*. *sojae* NBRC4239, and *A*. *luchuensis* NBRC4314 strains did not form sclerotia on the tested media, whereas their conidiation was also induced by copper (Supplementary Figures S5A,B). These results indicate that copper affects asexual development in *A*. *oryzae* industrial strains, *A*. *sojae*, and *A*. *luchuensis*.

### 3.3. Copper affects the expression of asexual development-related genes

**Figure 2:**
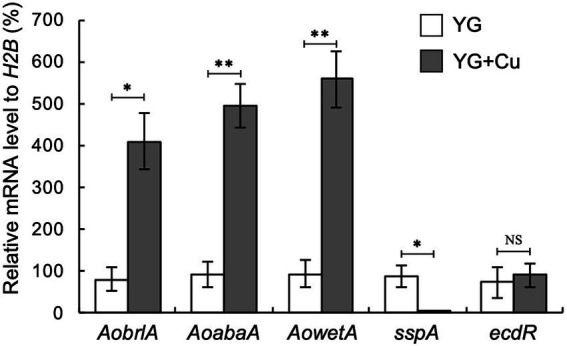
Copper stimulates expression of *AobrlA*, *AoabaA*, and *AowetA* and suppresses *sspA*. Conidial suspension (1 × 10^4^/5 μL) of the RIB40 strain was spotted onto YG and YG containing 1.6 μM CuSO_4_·5H_2_O (YG + Cu) and incubated in dark at 30°C for 5 days. Total RNA was extracted from collected mycelia, and then qRT-PCR for the indicated genes was performed. Data are shown as means ± SD of three independent experiments. *p < 0.05, **p < 0.01 by Student’s *t*-test. NS: Not significant.

Considering our observation that trace copper drastically changes asexual development, such as conidiation and sclerotia formation, we hypothesized that supplementation with copper might affect the expression of asexual development-related genes. In several *Aspergillus* species, activation of the central regulatory pathway, composed sequentially of *brlA*, *abaA*, and *wetA*, plays critical roles in conidiation (Park and Yu, 2012). *AobrlA*, *AoabaA*, and *AowetA* homologous to these genes are also required for proper conidiation in *A*. *oryzae* (Ogawa et al., 2010). The *sspA* gene, which is homologous to *ssp1* that encodes a major protein present in the mature sclerotia of *Sclerotinia sclerotium* (Li and Rollins, 2009), was upregulated in *A*. *oryzae* mutants that form an increased number of sclerotia (Jin et al., 2011a,b). In concordance with the developmental changes caused by copper, such as the induction of conidiation and inhibition of sclerotia formation, the mRNA levels of *AobrlA*, *AoabaA*, and *AowetA* were strongly upregulated, and that of *sspA* was downregulated by copper supplementation (Figure 2). These results suggest that trace copper affects the expression of the asexual development-related genes, resulting in induction of conidiation and inhibition of sclerotia formation.

### 3.4. AoBrlA has an inhibitory function in sclerotia formation

**Figure 3:**
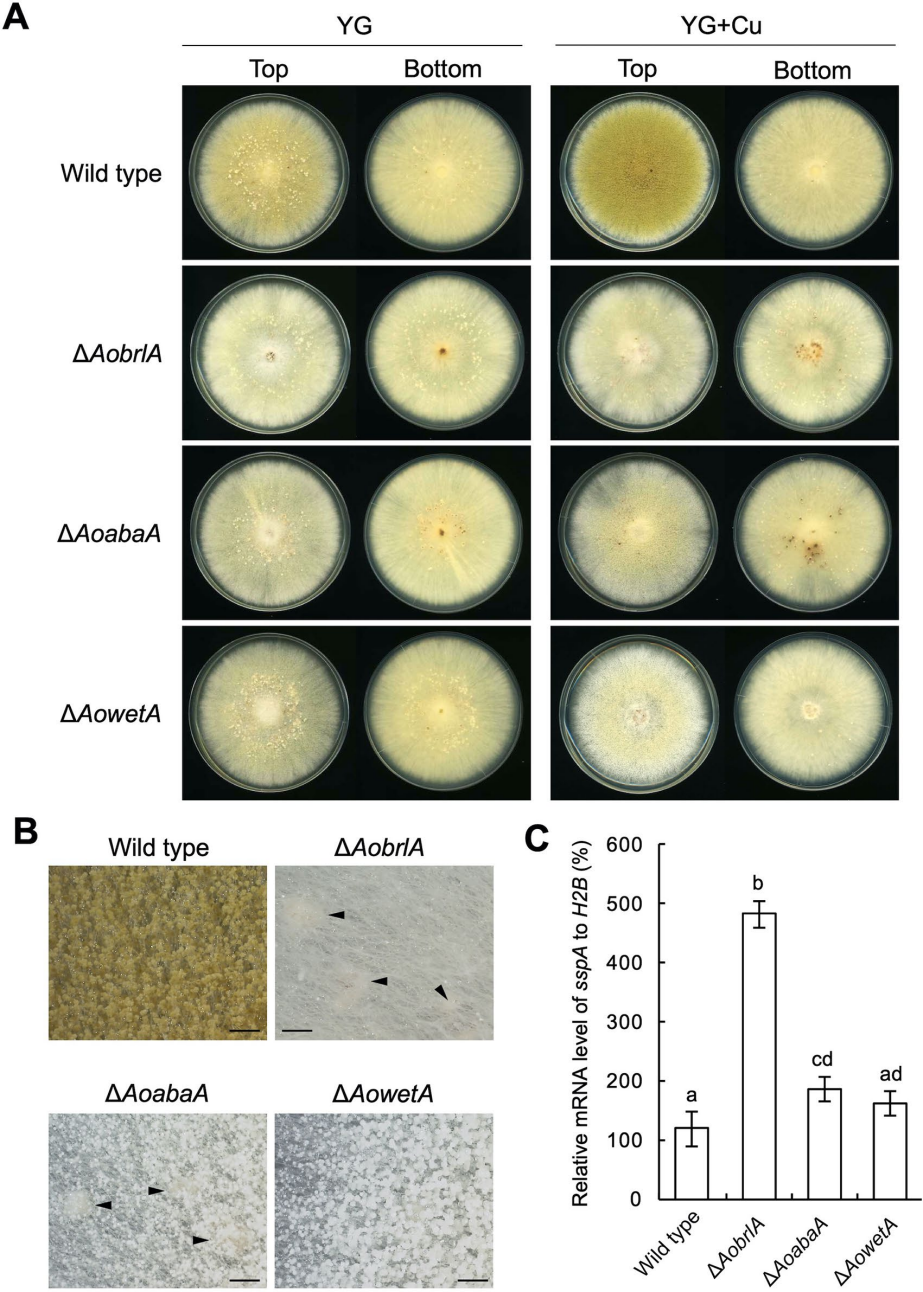
AoBrlA is required for the copper-mediated inhibition of sclerotia formation. **(A)** Conidial suspension (1 × 10^4^/5 μL) of the wild-type strain was spotted, and the mycelial mass of the indicated deletion strains was put onto YG and YG containing 1.6 μM CuSO_4_·5H_2_O (YG + Cu). They were incubated in dark at 30°C for 7 days. The top and bottom side pictures of the plates are shown as “Top” and “Bottom,” respectively. Sclerotia were detected as white or pigmented dots when observed from the bottom side of the plates. **(B)** Stereomicroscopic images of the colonies grown on YG containing 1.6 μM CuSO_4_·5H_2_O shown in panel A. Arrowheads indicate sclerotia. Bars: 1 mm. **(C)** The relative mRNA level of the *sspA* gene to that of *H2B*.The indicated strains were inoculated onto the YG agar media supplemented with 1.6 μM CuSO_4_·5H_2_O as described in panel **A** and incubated in dark at 30°C for 5 days. Data are shown as means ± SD of three independent experiments. Data are analyzed using Tukey’s multiple comparison test, and means sharing the same letter are not significantly different (*p* > 0.05).

Conidiation was induced and sclerotia formation was inhibited in the presence of copper (Figures 1D–F). In contrast, conidiation was reduced when sclerotia formation was induced (Figure 1D). Such phenotypes suggest a mutually exclusive relationship between conidiation and sclerotia formation. Additionally, the expression of *AobrlA*, *AoabaA*, and *AowetA* was downregulated when sclerotia formation was induced (Figure 2). Therefore, the expression of these genes might be involved in sclerotia formation. To investigate this possibility, sclerotia formation was examined in the deletion mutants of *AobrlA*, *AoabaA*, and *AowetA*. As speculated, the *AobrlA* and *AoabaA* deletion mutants showed increased numbers of sclerotia in the presence of copper, although induction of sclerotia formation was not detected in the *AowetA* deletion mutant (Figures 3A,B). According to this observation, the *sspA* gene was upregulated in the *AobrlA* and *AoabaA* deletion mutants (Figure 3C). However, as the increased level of *sspA* expression in the *AoabaA* deletion mutant was lower than that in the *AobrlA* deletion mutant (Figure 3C), AoBrlA but not AoAbaA functions as the main factor regulating the copper-mediated inhibition of sclerotia formation. Increased sclerotia formation in the *AobrlA* deletion mutant was suppressed by reintroducing the *AobrlA* gene (Supplementary Figure S6A). These results indicated that AoBrlA inhibit sclerotia formation.

### 3.5. EcdR is required for the developmental response caused by copper

**Figure 4:**
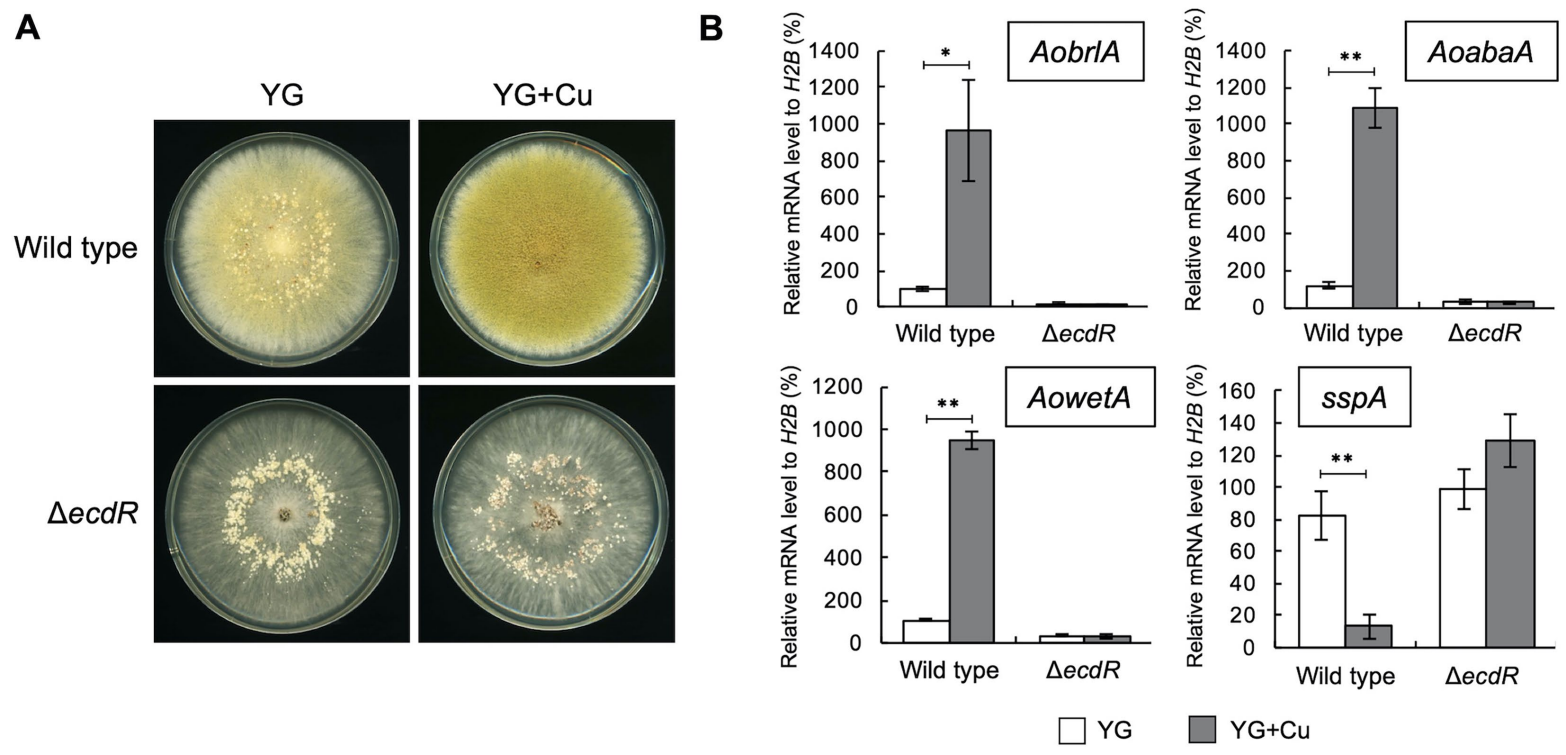
EcdR is required for the increased conidiation and decreased sclerotia formation in response to copper. **(A)** Conidial suspension (1 × 10^4^/5 μL) of the wild type strain was spotted and the mycelial mass of the *ecdR* deletion strain was put onto YG and YG containing 1.6 μM CuSO_4_·5H_2_O (YG + Cu). They were incubated in dark at 30°C for 7 days. **(B)** The relative mRNA levels of the indicated genes to that of *H2B*. Conidial suspension (1 × 10^4^/5 μL) of the wild type strain was spotted and the mycelial mass of the *ecdR* deletion strain was put onto YG and YG containing 1.6 μM CuSO_4_·5H_2_O (YG + Cu) and incubated in dark at 30°C for 5 days. Data are shown as means ± SD of three independent experiments. *p < 0.05, **p < 0.01 by Student’s *t*-test.

The transcription factor EcdR was previously suggested to be involved in the induction of *AobrlA*, and its deletion results in little conidiation and enhanced sclerotia formation (Jin et al., 2011a). Considering the inhibitory function of AoBrlA in sclerotia formation, EcdR may be involved in the inhibition of sclerotia formation by copper. As expected, the *ecdR* deletion mutant displayed numerous sclerotia and hardly formed conidia on the YG agar medium containing copper (Figure 4A). According to such developmental phenotypes, *AobrlA*, *AoabaA*, and *AowetA* were not induced, and *sspA* was not repressed in the *ecdR* deletion mutant grown on media supplemented with copper (Figure 4B). These results indicate that EcdR regulates the expression of *AobrlA* to inhibit sclerotia formation and induce conidiation in response to copper.

### 3.6. Copper-dependent superoxide dismutase is involved in the copper-mediated developmental regulation

As the presence of trace copper affects conidiation and sclerotia formation, we hypothesized that proteins related to copper homeostasis are involved in developmental regulation. As the copper-binding proteins with roles in copper homeostasis in *A*. *fumigatus* and yeasts have been previously listed (Wiemann et al., 2017), potential homologs to these copper-binding proteins were identified in the proteins encoded in the genome sequence of *A*. *oryzae*. In this study, copper transporters were not examined, but rather the focus was on determining the intracellular response to copper. The predicted copper-binding transcription factors AO090003000161 and AO090701001154 and a predicted Cu/Zn superoxide dismutase (SOD) AO090020000521 were identified by BLASTp analyses, and they were designated as AoAceA, AoMacA, and AoSod1, respectively (Table 1). Although proteins homologous to the copper-binding transcription factor CufA and the copper metallothionein CmtA of *A*. *fumigatus* were not found by BLASTp analysis, nucleotide sequences predicted to encode these proteins were found by tBLASTn analysis. The *cufA* homologous gene was predicted at Chr5:1,300,449-1,301,358 with two introns, and the *cmtA* homologous gene was predicted at Chr7:2,741,077-2,741,532 with two introns. Therefore, we designated these genes as *AocufA* and *AocmtA*, respectively (Table 1).

**Table 1:** The proteins involved in copper homeostasis in *A*. *oryzae*, *A*. *fumigatus*, and *S*. *cerevisiae*.

*Aspergillus oryzae*		*Aspergillus fumigatus*		*Saccharomyces cerevisiae*	Description
ID	Name	ID	Name	Name	
Copper-binding transcription factors					
AO090003000161	AoAceA	AfuA_6G07780	AceA	Ace1 (Cup2)	Copper-fist TF involved in copper detoxification
AO090701001154	AoMacA	AfuA_1G13190	MacA	Mac1 (Cua1)	Copper-fist TF involved in copper starvation
	AoCufA	AfuA_2G01190	CufA	Haa1	Copper-fist TF
Cu metallothioneins					
	AoCmtA	AfuA_4G04318	CmtA	Cup1	Copper metallothioneins
				Crs5	Copper metallothioneins
Superoxide dismutases					
AO090020000521	AoSod1	AfuA_5G09240	Sod1	Sod1	Cytoplasmic Cu/Zn-SOD

**Figure 5:**
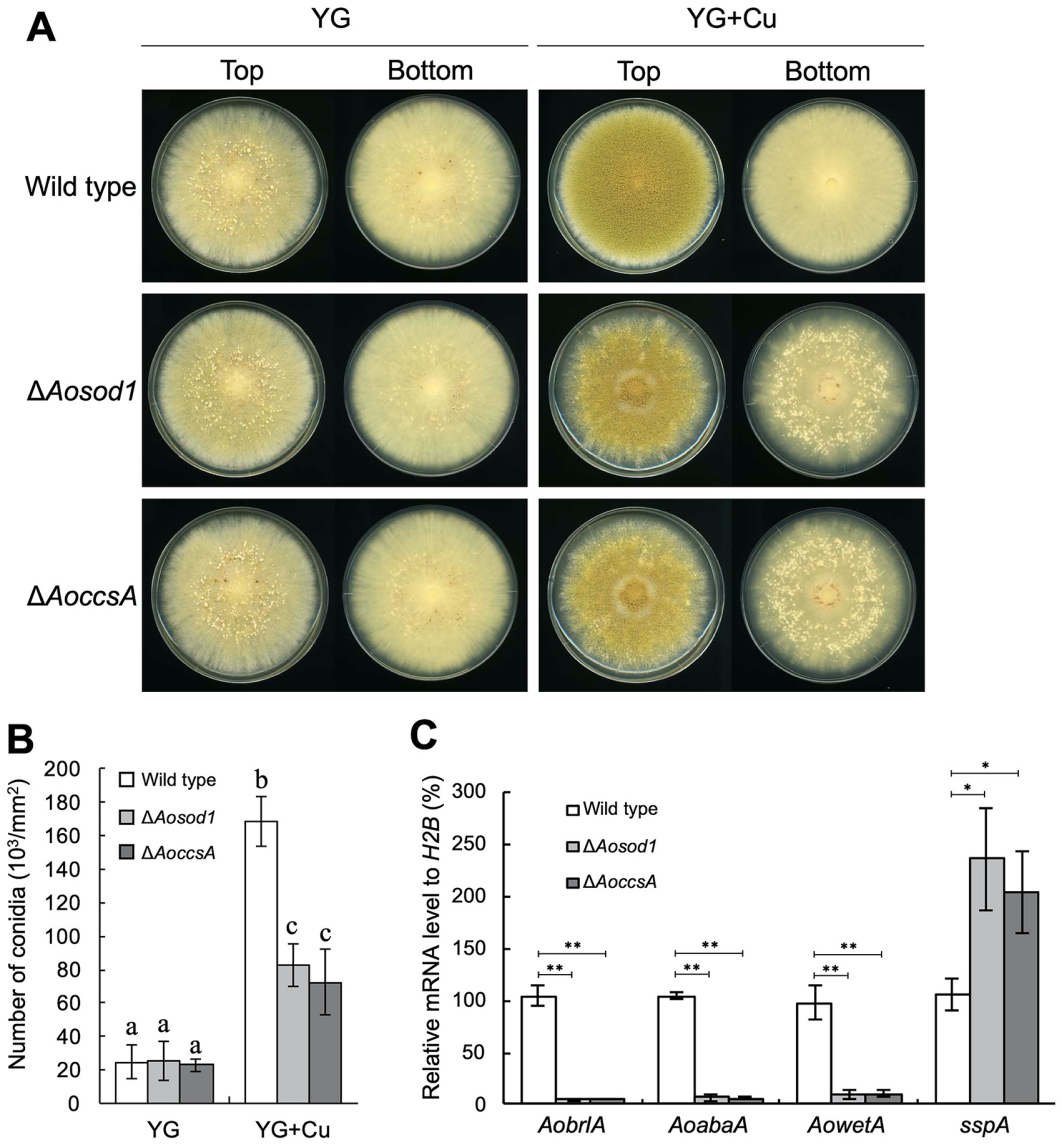
AoSod1 and AoCcsA are involved in copper-mediated developmental regulation. **(A)** Conidial suspension (1 × 10^4^/5 μL) of the indicated strains was spotted onto YG and YG containing 1.6 μM CuSO_4_·5H_2_O (YG + Cu) and incubated in dark at 30°C for 7 days. Sclerotia were detected as white or pigmented particles when observed from the bottom side of the plates. **(B)** Conidiation efficiencies of the *Aosod1* and *AoccsA* deletion mutants. Conidial suspensions (1 × 10^4^/5 μL) of the indicated strains were inoculated onto YG and YG containing 1.6 μM CuSO_4_·5H_2_O (YG + Cu), and then incubated in dark at 30°C for 7 days. Data are shown as means ± SD of three independent experiments. Data are analyzed using Tukey’s multiple comparison test, and means sharing the same letter are not significantly different (*p* > 0.05). **(C)** The relative mRNA levels of the indicated genes to that of *H2B*. Conidial suspension (1 × 10^4^/5 μL) of the indicated strains was spotted onto YG agar medium with 1.6 μM CuSO_4_·5H_2_O and incubated in dark at 30°C for 7 days. Data are shown as means ± SD of three independent experiments. *p < 0.05, **p < 0.01 by Student’s *t*-test.

To investigate the involvement of such copper-binding proteins in the copper-mediated regulation of asexual development, deletion mutants of the genes encoding them were constructed and incubated on YG agar medium with or without copper. As the *Aomac1* deletion mutant exhibited growth defects, its relationship with the developmental regulation was not investigated (Supplementary Figure S7). In the presence of copper, the *Aosod1* deletion mutant formed numerous sclerotia (Figure 5A), whereas the other deletion mutants did not form sclerotia (Supplementary Figure S7), suggesting that AoSod1 inhibits sclerotia formation. In *S*. *cerevisiae* and *A*. *fumigatus*, activation of Sod1 requires the copper chaperone Ccs1/CcsA, which introduces copper ions into Sod1 (Skopp et al., 2019; Du et al., 2021). Therefore, a copper chaperone for AoSod1 might be involved in the inhibition of sclerotia formation in *A*. *oryzae*. BLASTp analysis using the CcsA sequence of *A*. *fumigatus* showed AO090011000670 as its homologous protein in *A*. *oryzae*, which was designated as AoCcsA. The *AoccsA* deletion mutant formed numerous sclerotia in the presence of copper, as did the *Aosod1* deletion mutant (Figure 5A). In addition, the conidiation efficiencies of the *Aosod1* and *AoccsA* deletion mutants were reduced compared with that of the wild-type strain in the presence of copper (Figure 5B). Enhanced sclerotia formation and decreased conidiation of the *Aosod1* and *AoccsA* deletion mutants were suppressed by reintroducing these genes (Supplementary Figures S6B,C). These results indicate that AoSod1 and AoCcsA are involved in inhibition of sclerotia formation and induction of conidiation in response to copper. On the other hand, since conidiation was still induced by supplementation with copper in the *Aosod1* and *AoccsA* deletion mutants (Figure 5B), factors other than AoSod1 and AoCcsA may be involved in the induction of conidiation by copper.

To investigate how AoSod1 and AoCcsA mediate conidiation and sclerotia formation, a transcriptional analysis of *AobrlA*, *AoabaA*, *AowetA*, and *sspA* was performed under the condition of being supplemented with copper. In the *Aosod1* and *AoccsA* deletion mutants, the mRNA levels of *AobrlA*, *AoabaA*, and *AowetA* were downregulated (*p* < 0.01), and those of *sspA* were significantly induced (*p* < 0.05; Figure 5C). These results indicate that AoSod1 and AoCcsA affect the expression of genes involved in asexual development to induce conidiation and inhibit sclerotia formation in the presence of copper.

## 4. Discussion

In the present study, we found that trace copper regulates asexual development in *A*. *oryzae*. Induction of conidiation by copper was observed in the *A*. *oryzae* strains, as well as in *A*. *sojae* and *A*. *luchuensis* strains (Figure 1 and Supplementary Figure S5). Conidiation defects have been reported in deletion mutants of *Afmac1*, which encodes a copper-responsive transcription factor in *A*. *fumigatus* (Kusuya et al., 2017) and *Bcccc2*, which encodes a copper-transporting ATPase in *B*. *cinerea* (Saitoh et al., 2010). These findings suggest that copper induces conidiation in filamentous fungi. In contrast, copper strongly inhibited the sclerotia formation in *A*. *oryzae* (Figure 1). High concentrations of copper have also been reported to inhibit sclerotia formation in *A*. *oryzae* G15 strain (Long et al., 2017) and an *Aspergillus* strain (Rogers and Li, 1985). These findings indicate the inhibitory effects of copper on sclerotia formation in *A*. *oryzae*. In contrast to the inhibitory effects of copper on sclerotia formation, copper has been reported to induce sclerotia formation in *P*. *thomii* (Zhang et al., 2014; Zhao et al., 2014), and the *Bcccc2* deletion abolished sclerotia formation in *B*. *cinerea* (Saitoh et al., 2010). Therefore, the effects of copper on sclerotia formation vary in filamentous fungi.

**Figure 6:**
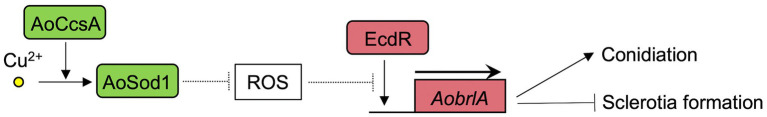
The predicted model of the asexual developmental regulation in response to copper in *A*. *oryzae*. The copper chaperone AoCcsA introduces copper into the superoxide dismutase AoSod1. AoSod1 activated by copper then scavenges intracellular ROS, which is predicted to interfere the *AobrlA* induction by EcdR as shown with the dashed line, resulting in induction of conidiation and inhibition of sclerotia formation.

The deletion mutant of *AobrlA*, which plays a critical role in conidiation, showed enhanced sclerotia formation and increased expression of *sspA* in the presence of copper (Figure 3), indicating that AoBrlA has inhibitory functions in sclerotia formation. The enhanced sclerotia formation in the *AobrlA* deletion mutant was suppressed by the reintroduction of *AobrlA*, supporting the inhibitory functions of AoBrlA in sclerotia formation. Additionally, in *A*. *flavus* which is genetically close to *A*. *oryzae*, deletion of *fluG* results in delayed induction of *brlA* and increased sclerotia formation (Chang et al., 2012), and *brlA* is downregulated under the sclerotial state (Wu et al., 2014). These findings also support the interpretation that AoBrlA has inhibitory functions in sclerotia formation in *A*. *oryzae*. However, it was previously reported that deletion of *AobrlA* did not enhance sclerotia formation under the condition where sclerotia formation was induced (Ogawa et al., 2010). Considering these findings, AoBrlA is thought to function as an inhibitor of sclerotia formation in the presence of trace copper but not in the condition inducing sclerotia formation (Figure 6). Moreover, the expression of *AobrlA* was induced in response to copper (Figure 2), suggesting that copper stimulates the expression of *AobrlA* to induce conidiation and inhibit sclerotia formation (Figure 6).

The *ecdR* deletion mutant formed a large number of sclerotia, even in the presence of copper (Figure 4A), indicating that EcdR is required for the copper-mediated inhibition of sclerotia formation. As EcdR was suggested to be an inducer of *AobrlA* at the early stage of conidiation (Jin et al., 2011a), it is suggested that copper stimulates transcriptional induction of *AobrlA* by EcdR to induce conidiation and inhibit sclerotia formation (Figure 6). However, it is unclear how copper affects induction of *AobrlA* by EcdR. As copper supplementation did not affect *ecdR* expression (Figure 2), copper-mediated developmental regulation does not seem to be dependent on a transcriptional change of *ecdR*.

The absence of cupper-dependent SOD AoSod1 and its copper chaperone AoCcsA inhibited conidiation and downregulated the expression of conidiation-related genes, such as *AobrlA*, *AoabaA*, and *AowetA* (Figure 5). In addition, deletion of *Aosod1* and *AoccsA* resulted in enhanced sclerotia formation in the presence of copper (Figure 5A), which is thought to be caused by the downregulation of *AobrlA*. SOD scavenges ROS (Staerck et al., 2017), and deletion of *sodA* and *ccsA* leads to increased ROS accumulation in *A*. *fumigatus* (Du et al., 2021). Therefore, it is suggested that copper may stimulate AoSod1 to scavenge intracellular ROS, and that intracellular ROS accumulation in the absence of AoSod1 or AoCcsA may inhibit *AobrlA* induction in *A*. *oryzae*, resulting in inhibited conidiation and an induction of sclerotia formation (Figure 6). The possible induction of sclerotia formation by ROS accumulation led to the speculation that oxidative stress affects sclerotia formation. However, treatment with oxidative stress-inducing agents did not stimulate sclerotia formation in the presence of copper (Supplementary Figure S8). This result is consistent with a previous report that treatment with the oxidative stress agent did not activate sclerotia formation (Papapostolou et al., 2014). Although the effects of copper and *Aosod1* deletion on ROS accumulation remain unclear, complex mechanisms besides ROS may be involved in the copper-mediated inhibition of sclerotia formation.

To date, the molecular mechanisms underlying changes in asexual development caused by environmental cues have not been well elucidated in *A*. *oryzae*. The present study demonstrating that trace copper mediates regulation of asexual development by inducing SOD and *AobrlA* will be greatly helpful in understanding such mechanisms in *A*. *oryzae*. As the involvements of these factors in sclerotia formation have not been reported, this study provides new insights into the regulatory mechanism of sclerotia formation. Therefore, our findings are expected to contribute to the enhancement of sclerotia formation and the ongoing search for sexual reproduction in *A*. *oryzae*.

## Data availability statement

The original contributions presented in the study are included in the article/Supplementary material, further inquiries can be directed to the corresponding author.

## Author contributions

TK and J-iM conceived and designed the research and wrote the manuscript. TK performed the experiments. All authors contributed to the article and approved the submitted version.

## Funding

This research was funded by Japan Society for the Promotion of Science (JSPS) KAKENHI grant number 17 K15242, Grant-in-Aid for Young Scientists (B), 20 K15429, Grant-in-Aid for Early-Career Scientists, 18H02123, Grant-in-Aid for Scientific Research (B), and 21H02098, Grant-in-Aid for Scientific Research (B).

## Conflict of interest

The authors declare that the research was conducted in the absence of any commercial or financial relationships that could be construed as a potential conflict of interest.

## Publisher’s note

All claims expressed in this article are solely those of the authors and do not necessarily represent those of their affiliated organizations, or those of the publisher, the editors and the reviewers. Any product that may be evaluated in this article, or claim that may be made by its manufacturer, is not guaranteed or endorsed by the publisher.
