# Supplementary material for: Trace copper-mediated asexual development via a superoxide dismutase and induction of AobrlA in Aspergillus oryzae
Source: Front Microbiol. 2023 Mar 8;14:1135012. doi: 10.3389/fmicb.2023.1135012 (PMC10030727; doi:10.3389/fmicb.2023.1135012)
Supplement: Supplementary file 1 [file Data_Sheet_1.PDF]

Supplementary Table 1. Strains used in this study

| Strain name | Host strain | Genotype/Description | Reference |
|---|---|---|---|
| *A. oryzae* strains | | | |
| RIB40 | | Wild | [Machida et al., 2005] |
| R40Δku5-2 | RIB40 | Δ*ku70* | This study |
| R40KS | R40Δku5-2 | Δ*ku70*, *sC*$^{-}$ | This study |
| R40KSN | R40KS | Δ*ku70*, *sC*$^{-}$, *niaD*$^{-}$ | This study |
| R40KN | R40KSN | Δ*ku70*, *niaD*$^{-}$ | This study |
| R40KSNP | R40KSN | Δ*ku70*, *sC*$^{-}$, *niaD*$^{-}$, Δ*pyroA* | This study |
| R40KSNΔecdR | R40KSNP | Δ*ku70*, *sC*$^{-}$, *niaD*$^{-}$, Δ*pyroA*, Δ*ecdR*::*pyroA* | This study |
| R40KSNΔAobrlA | R40KSNP | Δ*ku70*, *sC*$^{-}$, *niaD*$^{-}$, Δ*pyroA*, Δ*AobrlA*::*pyroA* | This study |
| R40KNΔAobrlA | R40KSNΔAobrlA | Δ*ku70*, *niaD*$^{-}$, Δ*pyroA*, Δ*AobrlA*::*pyroA* | This study |
| R40KN-AobrlA-com | R40KSNΔAobrlA | Δ*ku70*, *sC*$^{-}$, *niaD*$^{-}$, Δ*pyroA*, *AobrlA*::*sC*::*pyroA* | This study |
| R40KSNΔAoabaA | R40KSNP | Δ*ku70*, *sC*$^{-}$, *niaD*$^{-}$, Δ*pyroA*, Δ*AoabaA*::*pyroA* | This study |
| R40KSNΔAowetA | R40KSNP | Δ*ku70*, *sC*$^{-}$, *niaD*$^{-}$, Δ*pyroA*, Δ*AowetA*::*pyroA* | This study |
| R40KSNΔAosod1 | R40KSNP | Δ*ku70*, *sC*$^{-}$, *niaD*$^{-}$, Δ*pyroA*, Δ*Aosod1*::*pyroA* | This study |
| R40KSΔAosod1 | R40KSΔAosod1 | Δ*ku70*, *sC*$^{-}$, Δ*pyroA*, Δ*Aosod1*::*pyroA* | This study |
| R40KS-Aosod1-com | R40KSΔAosod1 | Δ*ku70*, *sC*$^{-}$, *niaD*$^{+}$::*Aosod1*, Δ*pyroA*, Δ*Aosod1*::*pyroA* | This study |
| R40KSNΔAoccsA | R40KSNP | Δ*ku70*, *sC*$^{-}$, *niaD*$^{-}$, Δ*pyroA*, Δ*AoccsA*::*pyroA* | This study |
| R40KSΔAoccsA | R40KSNΔAoccsA | Δ*ku70*, *sC*$^{-}$, Δ*pyroA*, Δ*AoccsA*::*pyroA* | This study |
| R40KS-AoccsA-com | R40KSNΔAoccsA | Δ*ku70*, *sC*$^{-}$, *niaD*$^{+}$::*AoccsA*, Δ*pyroA*, Δ*AoccsA*::*pyroA* | This study |
| R40KSNΔAomacA | R40KSNP | Δ*ku70*, *sC*$^{-}$, *niaD*$^{-}$, Δ*pyroA*, Δ*AomacA*::*pyroA* | This study |
| R40KSNΔAoaceA | R40KSNP | Δ*ku70*, *sC*$^{-}$, *niaD*$^{-}$, Δ*pyroA*, Δ*AoaceA*::*pyroA* | This study |
| R40KSNΔAocufA | R40KSNP | Δ*ku70*, *sC*$^{-}$, *niaD*$^{-}$, Δ*pyroA*, Δ*AocufA*::*pyroA* | This study |
| R40KSNΔAocmtA | R40KSNP | Δ*ku70*, *sC*$^{-}$, *niaD*$^{-}$, Δ*pyroA*, Δ*AocmtA*::*pyroA* | This study |
| TK-32 | | For sake fermentation | [Watarai et al., 2019] |
| TK-38 | | For sake fermentation | [Watarai et al., 2019] |
| TK-48 | | For soy sauce fermentation | [Watarai et al., 2019] |
| *A. sojae* strain | | | |
| NBRC4239 | | For soy sauce fermentation | [Sato et al., 2011] |
| *A. luchuensis* strain | | | |
| NBRC4314 | | For awamori fermentation | [Yamada et al., 2016] |

Supplementary Table 2. Primers used in this study

| Primer name | Sequence (5′ to 3′) |
|---|---|
| Deletion of *ku70* | |
| SmaIIF1-PU6-F2nd | TCGAGCTCGGTACCCTTTTA |
| gku70-PU6R | TTCTAGCTCTAAAACCAGTGACTTCATCTCTTCGGGCACTTGTTCTTCTTTACAATGATTTATTT |
| 19IF-ku70-5F | CGGTACCCGGGGATCGGGTTTATGAGGCGCACTTT |
| ku70-5R | GCGTTGTCTGTGAATGTGTTGAGAGTCGTA |
| ku70-3F | CATTCACAGACAACGCTAGTATTGGTTACG |
| 19IF-ku70-3R | CGACTCTAGAGGATCCAAGGCGGATCATCGCATCA |
| | |
| Deletion of 3′ part of *niaD* | |
| DniaD-F | CCGCAGGGTTACCAGGGTAGAAATA |
| DniaD-R | CCTGCCCATACTGAAGCGAGTGATA |
| | |
| Deletion of *pyroA* | |
| gpyroA-PU6R | TTCTAGCTCTAAAACGGGCTCTGATATCGGCGGGGACACTTGTTCTTCTTTACAATGATTTATTT |
| 19IF-pyroA5F | CGGTACCCGGGGATCGGTAGAACGGCCAATGTAAAG |
| pyrG-pyroA5R | AATAGTCCTCTCGGGCCACTCGAGTGTGAGAGAGGTTTGTCCGA |
| pyrG-pyroA3F | AGCCTGGGGCACATTACCTCGAGATACCCCATTTGCTGTTTCGT |
| 19IF-pyroA3R | CGACTCTAGAGGATCGGGAGTGGTGTTCGGTCATT |
| pyrGF | TGGCCCGAGAGGACTATT |
| pyrGR | GGTAATGTGCCCCAGGCT |
| | |
| Amplification of the *pyroA* marker | |
| AopyroAF | CATATTTGGGCATGCAGCG |
| AopyroAR | GCCGAAGTCCTATCGGCAAA |
| | |
| Deletion of *ecdR* | |
| ecdR-1 | CTCGGTACCCGGGGACCCGACAGGAGGTTCCATCA |
| ecdR-2 | CATGCCCAAAATATGATCTTCAAACACCACGGCTG |
| ecdR-3 | CGATAGGACTTCGGCGCTGGTGGCAGGGCATGTTG |
| ecdR-4 | GTCGACTCTAGAGGAGCAAGAGGTGAATGCGTGTC |
| | |
| Deletion of *AobrlA* | |
| brlA-1 | CTCGGTACCCGGGGACCCCAAGAGTCATTCTTCAT |
| brlA-2 | CATGCCCAAAATATGCTTCACTGTGTCGAGGTTCA |
| brlA-3 | CGATAGGACTTCGGCGGATATTACGGGTGTATCCCT |
| brlA-4 | GTCGACTCTAGAGGAGTTCCGCCAATAGGGACAAT |
| | |
| Complementation of *AobrlA* | |
| sC-brlA-R | TGCGAGATCGGATCGCCATGCGTCAGTAGCCATCT |
| sC-F | CGATCCGATCTCGCAGATGT |
| pyroA-sC-R | CATGCCCAAAATATGCCGCGGATTTTCTGAGATTT |
| 19IF-pyroAmid-R | GTCGACTCTAGAGGACTACCTGGCATTCAACGAAA |
| | |
| Deletion of *AoabaA* | |
| abaA-1 | CTCGGTACCCGGGGAGCAGATGGACCGGATGAAAC |
| abaA-2 | CATGCCCAAAATATGGGAGCGGACCACCTCGAATA |
| abaA-3 | CGATAGGACTTCGGCCGCTGGGTTTCGGGACTTTT |
| abaA-4 | GTCGACTCTAGAGGATGCCGCTCTCCGAGTTCCAT |
| | |
| Deletion of *AowetA* | |
| wetA-1 | CTCGGTACCCGGGGAGCGCATTCCTCTCCAGCTTT |
| wetA-2 | CATGCCCAAAATATGGCGGCGATGGTGGTTCCAAG |

| | |
|---|---|
| wetA-3 | CGATAGGACTTCGGCCTGGAGGTAATCCCGTTTGT |
| wetA-4 | GTCGACTCTAGAGGACCAGCAACGCACATGAAAAT |
| Deletion of *AomacA* | |
| mac1-1 | CTCGGTACCCGGGGACATACCTGCCATTTGAGACA |
| mac1-2 | CATGCCCAAAATATGCTGTGCCTCGTGCTGTTCTG |
| mac1-3 | CGATAGGACTTCGGCCCCCATCTCGAAATACCTTG |
| mac1-4 | GTCGACTCTAGAGGAGGCAAGCTCTGGTCTTTTTG |
| Deletion of AoaceA | |
| 003_0161-1 | CTCGGTACCCGGGGACATCCTTGGACCTTGAATTG |
| 003_0161-2 | CATGCCCAAAATATGCGCGGTTACTGGGTAGCAAA |
| 003_0161-3 | CGATAGGACTTCGGCCGCGGCAAGCGCAGGTTTT |
| 003_0161-4 | GTCGACTCTAGAGGAGTACGTGGGTTGCGTGGTAT |
| Deletion of *Aosod1* | |
| 020_0521-1 | CTCGGTACCCGGGGACCATGCAATGTCCTCATGTA |
| 020_0521-2 | CATGCCCAAAATATGTTTGACGGTTGGGGGTTAAT |
| 020_0521-3 | CGATAGGACTTCGGCTGTGAAGTGCCGGGTCAGAA |
| 020_0521-4 | GTCGACTCTAGAGGACGCTAATGTCGGGCAACAAA |
| Complementation of *Aosod1* | |
| UXN-020_0521F | ACGAGCTGGGAACTCGGCCGGTGCCTACCTCATCT |
| UXN-020_0521R | TATTAAAGAATACTCGAACCAGTGTGGGAGACAAT |
| Deletion of *AoccsA* | |
| 011_0670-1 | CTCGGTACCCGGGGAGACCTGGGAGAGGAGAGTTT |
| 011_0670-2 | CATGCCCAAAATATGGATGTACGTTTGGGACGTGT |
| 011_0670-3 | CGATAGGACTTCGGCGGGCATCTATTTGATGCTCC |
| 011_0670-4 | GTCGACTCTAGAGGAGGGAGTACCAATAGATCCGA |
| Complementation of *AoccsA* | |
| UXN-011_0670F | ACGAGCTGGGAACTCCTGGGAGAGGAGAGTTTTGA |
| UXN-011_0670R | TATTAAAGAATACTCTCCAGTCCATCCCTCACTTT |
| Deletion of *AocmtA* | |
| AocmtA-1 | CTCGGTACCCGGGGACGAGATGTTACATTGGCGTT |
| AocmtA-2 | CATGCCCAAAATATGTGTGAATTCTCGTCGATTTG |
| AocmtA-3 | CGATAGGACTTCGGCCTCGGTTGCTATTTTGTCTTG |
| AocmtA-4 | GTCGACTCTAGAGGAGCCGTCCTTCCTCACTATTT |
| Deletion of *AocufA* | |
| AocufA-1 | CTCGGTACCCGGGGACTCAGCCACCTCCATTCATT |
| AocufA-2 | CATGCCCAAAATATGTCCTTGAGGAGTATGAGCGG |
| AocufA-3 | CGATAGGACTTCGGCGTTGGGGGGTGGGGGGATGA |
| AocufA-4 | GTCGACTCTAGAGGAGGAGGAGCCGACGAAATAAC |
| Primers for genomic PCR | |
| niaD-GEF | CCGTATGTCTAAAGGGCACTTATTT |
| niaD-cP-F | GCCGCAGGGTTACCAGGGTAGAAAT |
| niaD-cP-R | GCGGTGATATTGATGGCACGATAGA |
| ssC-coPF | CCGTTGGGTGTCTTGCAGATTACTT |
| sC-GER | CCCGCGGATTTTCTGAGATTTAGTT |
| pyroA-cPF | GGGCTTTGCGCGTCAGTTTCACAG |

| | |
|---|---|
| pyroA-3outR | GGGGGGGTTCTTGGTCTCCTTCTT |
| pyroA-mid-CoPR | TAGATGGCGACAACCACGATCAAAA |
| ecdR-5 | GGGCCTCGCGTCTGTGCCCATTTTG |
| ecdR-6 | CCAAGGCATCCCGGTGTCAATAATC |
| brlA-5 | CGCTATGCTTGCTATGCTATGCTTT |
| brlA-6 | CCCCATGCGTCAGTAGCCATCTAAA |
| abaA-5 | GGCTATCGATGCGGAGCAAGAAAT |
| abaA-6 | CCCCGTAAACTCCTATGCCAGAAAC |
| wetA-5 | GCACCTGCCGCGCATATCTTTTGTT |
| wetA-6 | CGGCCCGGGAGGGCTGATTAAGATG |
| mac1-5 | GCCATTCGGGTCAGAGGCTATAAGA |
| mac1-6 | CGGCCGAAGATGAAACGCGGTGTAA |
| 003_0161-5 | CGGGGTGCATGACAAGTCCTCACTA |
| 003_0161-6 | TAACGTGCCGCGAACAGACCACAAA |
| 020_0521-5 | GAAGCTGCGTGAGGGTATCTCCAA |
| 020_0521-6 | GTCCTCCAGGAACCGCATAGAAGAT |
| 011_0670-5 | CGCCCCGGTTGTTCATCAAAAGATT |
| 011_0670-6 | GCTCCCGCCTCTCCAGTCACAAAGT |
| AocmtA-5 | CTTGGCGTTCGCATCTTCATTCCAG |
| AocmtA-6 | CCAACCCAACGCCCTATAAGAACTC |
| AocufA-5 | GCTGCTAGTACTCGGGCTGCTAATC |
| AocufA-6 | AAGAGGGAAAAGAACCCACCCAATA |
| | |
| Primers for qRT-PCR | |
| brlA-RTF | ACTTTTGGCTTCCTTCGGAT |
| brlA-RTR | TACTGCGAACCATAGGAGGG |
| abaA-RTF | CGAGAGACTACTTCCGCACC |
| abaA-RTR | GGCCATTCTTTTGCTGTCAT |
| wetA-RTF | GCTGATGATCGATTCCCACT |
| wetA-RTR | AGTCTCCCGAATGATGGATG |
| sspA-RTF | TGCTACGGTGAATAATGGCA |
| sspA-RTR | GTGTTCCGGGATAAAAGGGT |
| ecdR-RTF | TCGCCCCAAAGCATATTAAC |
| ecdR-RTR | CTCCCTCTGGTCGTCACATT |

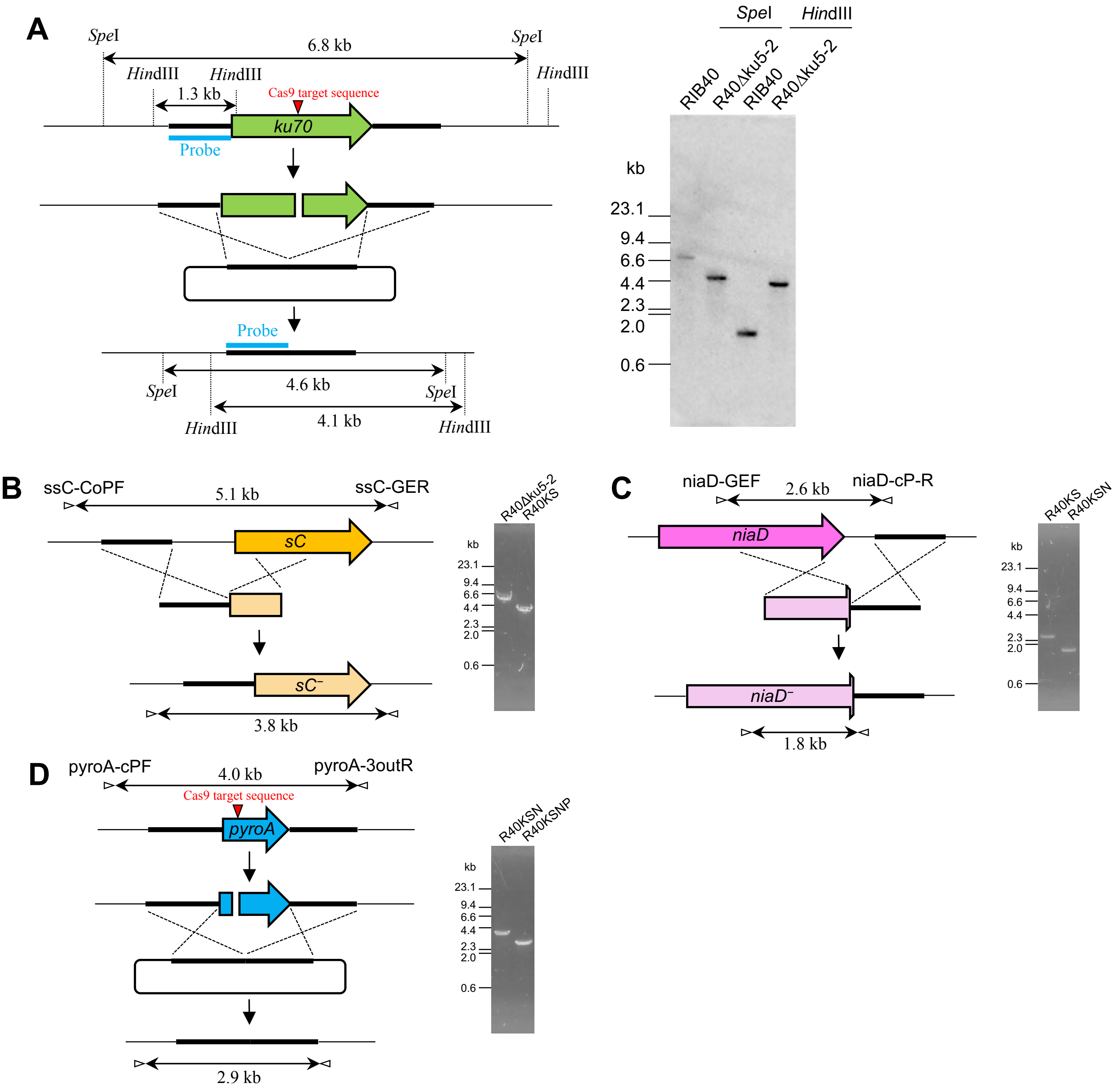


Supplementary Figure 1. Construction of the Δ*ku70* strain with *sC*⁻, *niaD*⁻, and Δ*pyroA*.
(A) Scheme for *ku70* deletion by genome editing. Genomic DNAs were digested with *Hin*dIII or *Spe*I, and the DNA fragment amplified using primers 19-IF-ku70-5F/ku70-5R was used as a probe for Southern blot analysis. (B, C) Schemes for constructiosn of the *sC*⁻ strain (B) and the *niaD*⁻ strain (C). (D) Scheme for *pyroA* deletion by genome editing.

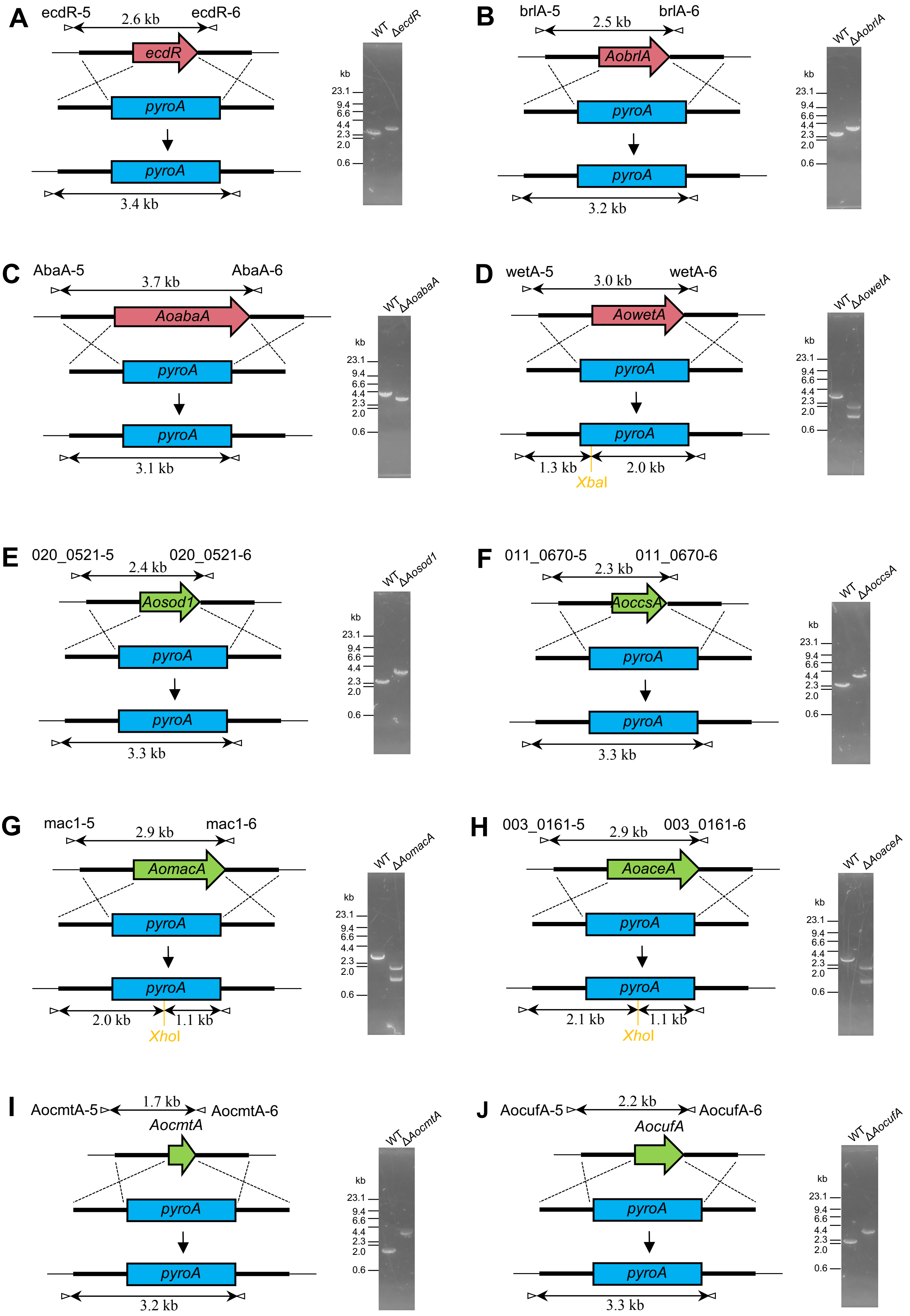


Supplementary Figure 2. Construction of the gene deletion mutants.
Schemes for *ecdR* deletion (A), *AobrlA* deletion (B), *AoabaA* deletion (C), *AowetA* deletion (D), *Aosod1* deletion (E), *AoccsA* deletion (F), *AomacA* deletion (G), *AoaceA* deletion (H), *AocmtA* deletion (I), and *AocufA* deletion (J).

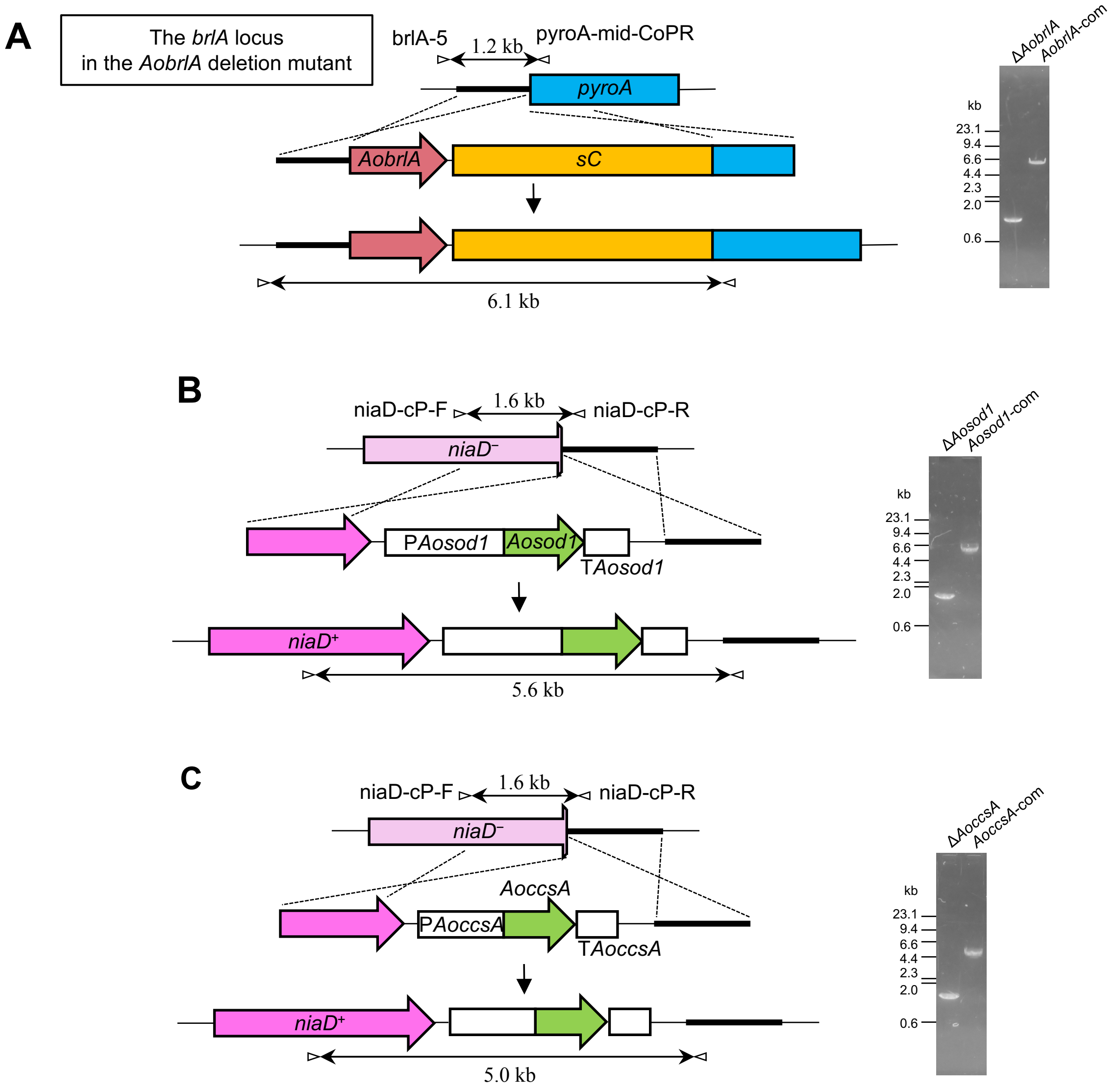


Supplementary Figure 3. Construction of the complemented strains.
(A) Scheme for reintroducing *AobrlA* into its native locus in the *AobrlA* deletion mutant, where the *AobrlA* ORF was replaced with the *pyroA* marker. (B, C) Schemes for reintroducing *Aosod1* into the *Aosod1* deletion mutant (B) and *AoccsA* into the *AoccsA* deletion mutant.

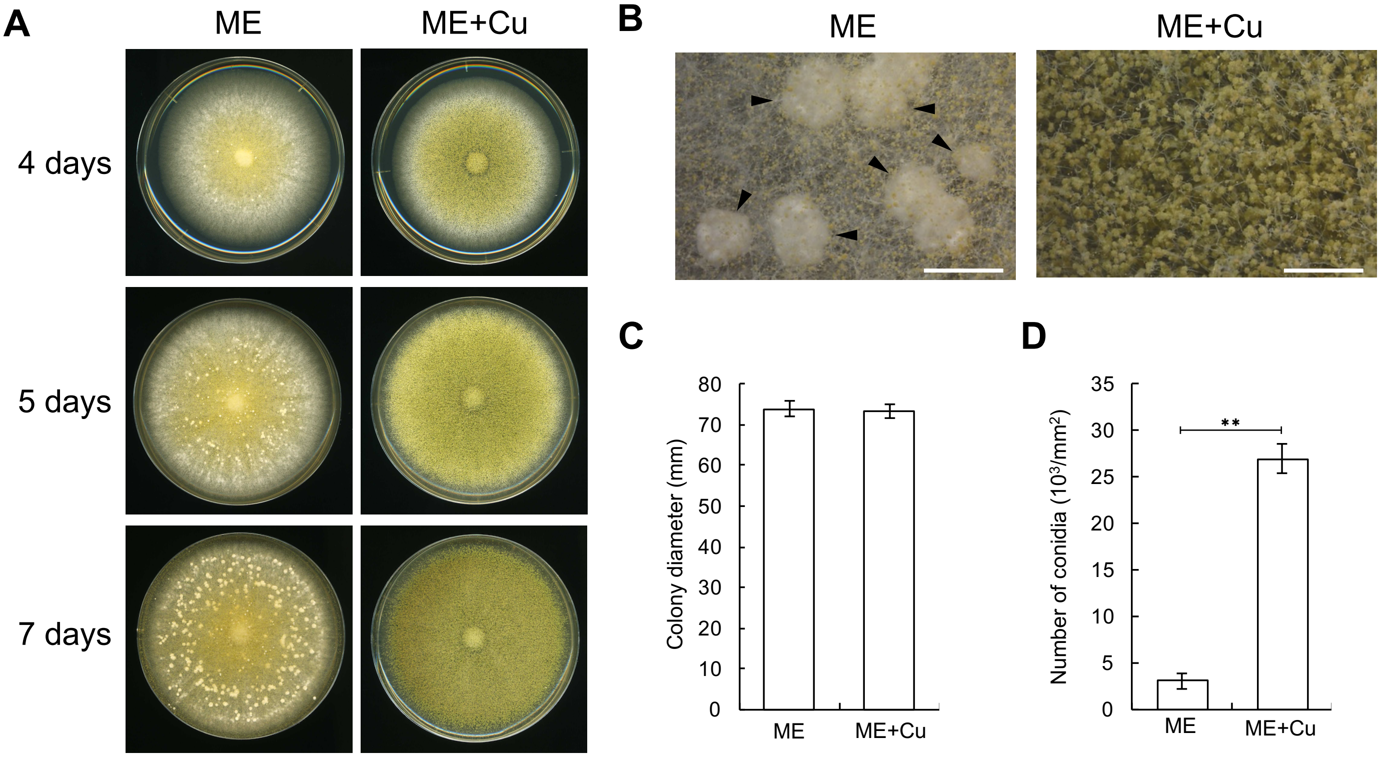


Supplementary Figure 4. Effects of copper supplementation to the ME agar medium on sclerotia formation and conidiation.

(A) Conidial suspensions ($1\times10^4$/5 µl) of the RIB40 strain were inoculated onto ME and ME containing 1.6 µM $CuSO_4{\cdot}5H_2O$ (ME+Cu) and incubated at 30°C for 4, 5, or 7 days. (B) Stereomicroscopic images of the colonies shown as 7 days in panel A. Arrowheads indicate sclerotia. Bars: 2 mm. (C, D) Effect of $CuSO_4{\cdot}5H_2O$ on the growth and conidiation efficiency. Colony diameter (C) and number of conidia (D) were measured under the condition shown as 5 days in panel A. The RIB40 strain was incubated on the ME and ME containing 1.6 µM $CuSO_4{\cdot}5H_2O$ (ME+Cu). Data are shown as means ± S.D. of three independent experiments. **$p < 0.01$ by Student's *t*-test.

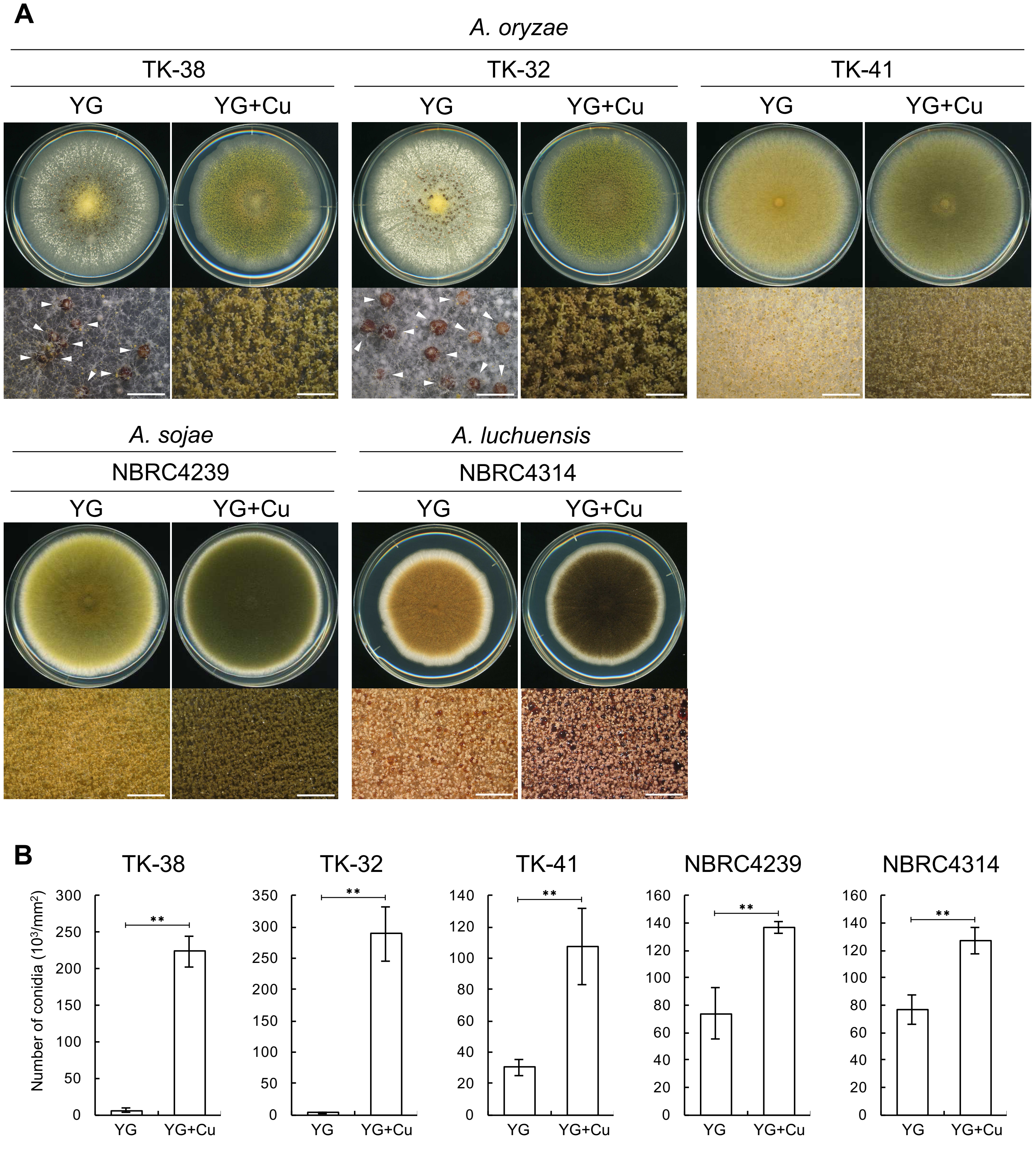


Supplementary Figure 5. Effects of copper supplementation on sclerotia formation and conidiation in *A. oryzae*, *A. sojae*, and *A. luchuensis* industrial strains

(A) Conidial suspensions ($1\times10^4$/5 µl) of the indicated strains were inoculated onto YG and YG containing 1.6 µM $CuSO_4\cdot5H_2O$ (YG+Cu) and incubated at 30°C for 7 days. Upper images indicate colonies and lower images indicate the results of the stereomicroscopy. Arrowheads indicate sclerotia. Bars: 2 mm. (B) Number of conidia in the indicated strains. Conidial suspensions ($1\times10^4$/5 µl) of the indicated strains were inoculated onto YG and YG containing 1.6 µM $CuSO_4\cdot5H_2O$ (YG+Cu) and incubated at 30°C for 5 days. Data are shown as means ± S.D. of three independent experiments. ** $p <$ 0.01 by Student's *t*-test.

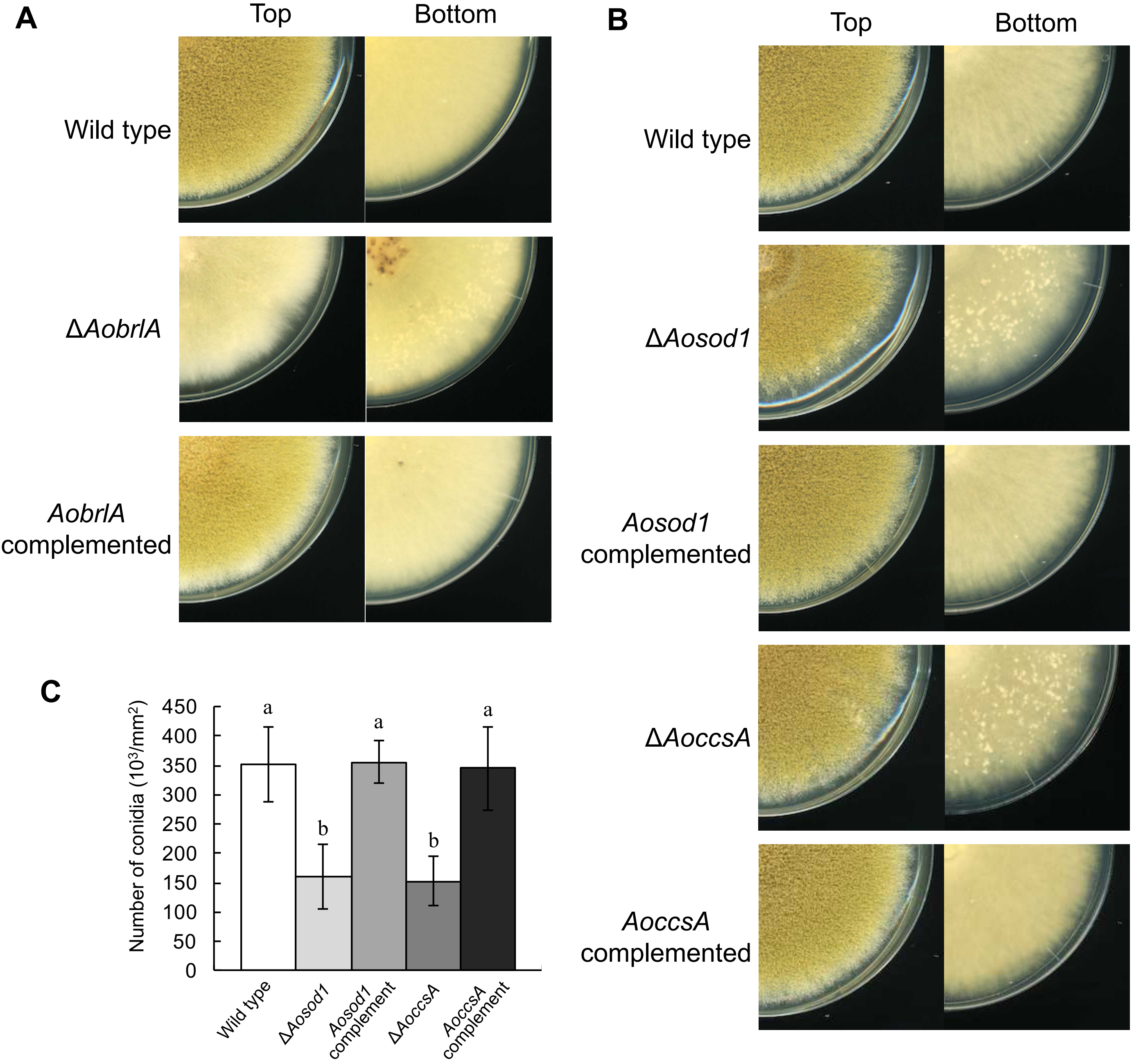


Supplementary Figure 6. Sclerotia formation of the *AobrlA*, *Aosod1*, and *AoccsA* complemented strains.

(A) Conidial suspensions ($1\times10^4$/5 µl) of the indicated strains were inoculated onto YG agar medium with 1.6 µM $CuSO_4 \cdot 5H_2O$ and incubated at 30°C for 7 days. (B) Conidial suspensions ($1\times10^4$/5 µl) of the indicated strains were inoculated onto YG agar medium with 1.6 µM $CuSO_4 \cdot 5H_2O$ and incubated at 30°C for 7 days. (C) Number of conidia of the indicated strains under the condition described in panel B. Data are shown as means ± S.D. of three independent experiments. Data are analyzed using Tukey's multiple comparison test, and means sharing the same letter are not significantly different ($p > 0.05$).

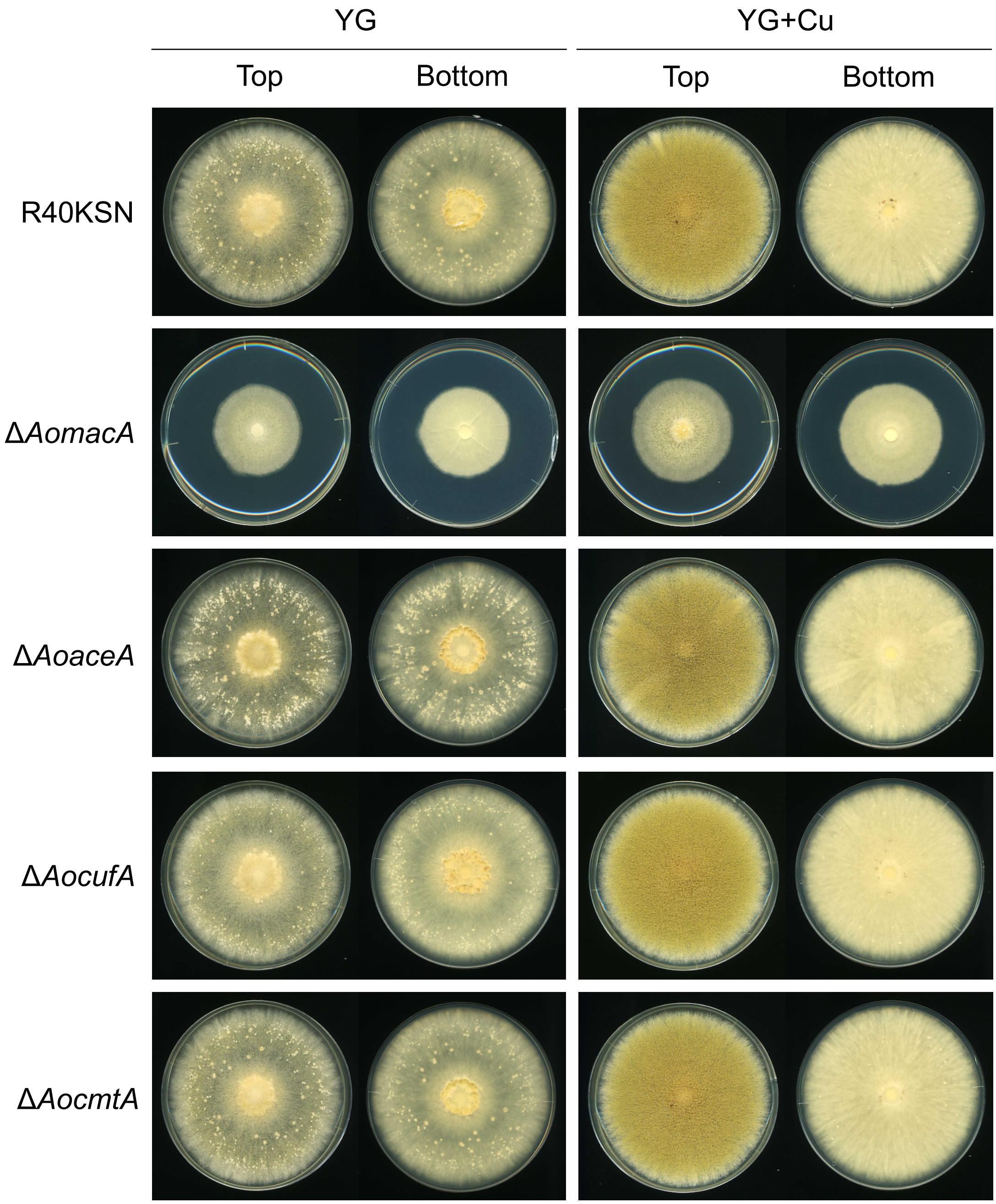


Supplementary Figure 7. Sclerotia formation in deletion mutants of the copper homeostasis-related genes

Conidial suspensions ($1\times10^4$/5 μl) of the indicated strains were inoculated onto YG and YG containing 1.6 μM $CuSO_4 \cdot 5H_2O$ (YG+Cu) and incubated at 30°C for 7 days.

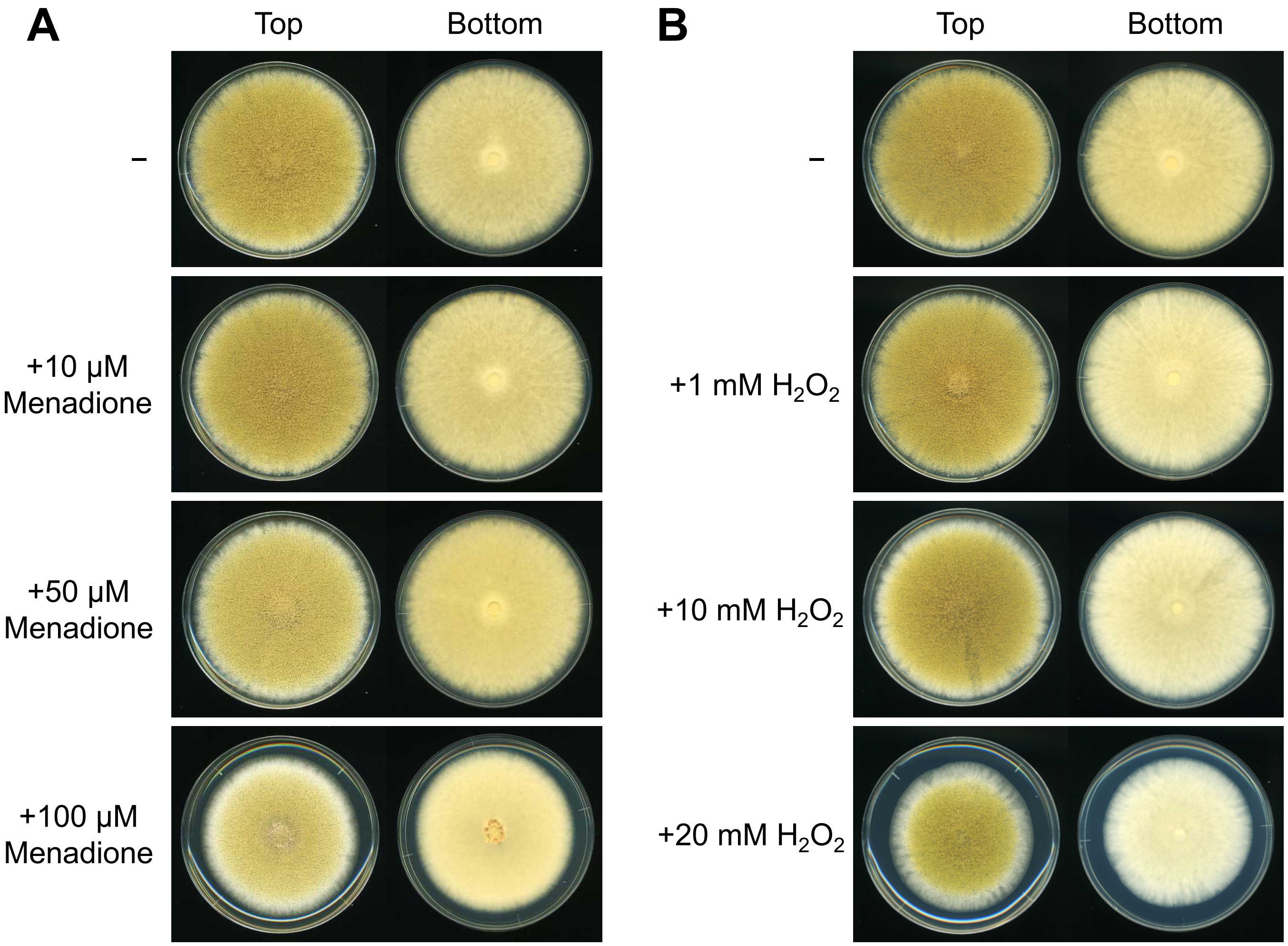


Supplementary Figure 8. Effect of oxidative stress on sclerotia formation

Conidial suspensions ($1\times10^4$/5 μl) of the RIB40 strain were inoculated onto YG agar media containing 1.6 μM $CuSO_4\cdot5H_2O$ with menadione (A) or $H_2O_2$ (B) and incubated at 30°C for 7 days.
